# A General Waveguide Circuit Theory

**DOI:** 10.6028/jres.097.024

**Published:** 1992

**Authors:** Roger B. Marks, Dylan F. Williams

**Affiliations:** National Institute of Standards and Technology, Boulder, CO 80303

**Keywords:** characteristic impedance, circuit theory, microwave measurement, network analyzer, pseudo-waves, reciprocity, reference impedance, transmission line, traveling waves, waveguide

## Abstract

This work generalizes and extends the classical circuit theory of electromagnetic waveguides. Unlike the conventional theory, the present formulation applies to all waveguides composed of linear, isotropic material, even those involving lossy conductors and hybrid mode fields, in a fully rigorous way. Special attention is given to distinguishing the traveling waves, constructed with respect to a well-defined characteristic impedance, from a set of pseudo-waves, defined with respect to an arbitrary reference impedance. Matrices characterizing a linear circuit are defined, and relationships among them, some newly discovered, are derived. New ramifications of reciprocity are developed. Measurement of various network parameters is given extensive treatment.

## 1. Introduction

Classical waveguide circuit theory, of which Refs. [1,2,3,4] are representative, proposes an analogy between an arbitrary linear waveguide circuit and a linear electrical circuit. The electrical circuit is described by an *impedance matrix*, which relates the normal electrical currents and voltages at each of its terminals, or ports. The waveguide circuit theory likewise defines an impedance matrix relating the *waveguide voltage* and *waveguide current* at each port. In both cases, the characterization of a network is reduced to the characterization of its component circuits. The primary caveat of waveguide circuit theory is that, at each port, a pair of identical waveguides must be joined without discontinuity and must transmit only a single mode, or at most a finite number of modes.

A great deal of confusion regarding waveguide circuits arises from the tendency to overemphasize the analogy to electrical circuits. In fact, important differences distinguish the two. For instance, the waveguide voltage and current, in contrast to their electrical counterparts, are highly dependent on definition and normalization. Also, the general conditions satisfied by the impedance matrix are different in the two cases. Furthermore, only the waveguide circuits, not electrical ones, are describable in terms of traveling waves. The latter two distinctions have been particularly neglected in the literature. In this introduction, we discuss all three of these differences and their relationship to the general waveguide circuit theory.

All waveguide circuit theories are based on some defined waveguide voltage and current. These definitions rely upon the electromagnetic analysis of a single, uniform waveguide. Eigenfunctions of the corresponding electromagnetic boundary value problem are waveguide modes which propagate in either direction with an exponential dependence on the axial coordinate. When limited to a single mode, the field distribution is completely described by a pair of complex numbers indicating the complex intensity (amplitude and phase) of these two counterpropagating *traveling waves.* The waveguide voltage and current, which are related to the electric and magnetic Fields of the mode, are linear combinations of the two traveling wave intensities. This linear relationship depends on the *characteristic impedance* of the mode.

The classical definition of the waveguide voltage and current is suitable only for modes which are TE (transverse electric), TM (transverse magnetic), or TEM (transverse electromagnetic). This includes many conventional waveguides, such as lossless hollow waveguide and coaxial cable. However, modes of guides with transversely nonuniform material parameters are generally hybrid rather than TE, TM, or TEM. Thus, the classical theory is inapplicable to multiple-dielectric guides, such as microstrip, coplanar waveguide, and optical fiber waveguide. Neither does it apply to lines containing an imperfect conductor, for a lossy conductor essentially functions as a lossy dielectric. This limitation has become increasingly important with the proliferation of miniature, integrated-circuit waveguides, in which the loss is a nonnegligible factor.

In the absence of a general theory, the most popular treatment of arbitrary waveguides is based on an engineering approach (for example, Ref. [5]). The procedure makes use of the fact that, in TE, TM, and TEM modes, the conventional waveguide voltage and current obey the same telegrapher’s equations which govern propagation in a low-frequency transmission line. The characteristic impedance, which enters the telegrapher’s equations, can be written in terms of equivalent circuit parameters *C, G, L*, and *R* Engineers assume that waveguide voltages and currents satisfying the telegrapher’s equations continue to exist for hybrid and lossy modes. Heuristic arguments, based on low-frequency circuit theory, are used to compute the equivalent circuit parameters, and those parameter estimates are used to determine the characteristic impedance from the conventional expression.

In fact, a practical, general definition of waveguide voltage *v* and current *i* is easily constructed using methods analogous to those applied to ideal TE, TM, and TEM modes. The basic principle [1, pp. 76-77] is that, for consistency with electrical circuit theory, *v* and *i* should be related to the complex power *p* by p=vi*. This ensures that *v* and *I* are proportional to the transverse electric and magnetic fields. Reference [1] declines to further specify *v* and *i*, arguing that their ratio *v*/*i* is irrelevant and arbitrary. In fact, *v*/*i* is often pertinent. When only the forward-propagating mode exists, then *v*/*i* =*Z*_0_, the characteristic impedance. As pointed out by Brews [6], *Z*_0_ is not entirely arbitrary; the relationship p = vi* determines the phase of *v*/*i* and therefore of *Z*_0_. The magnitude of *Z*_0_ is formally arbitrary, but its normalization plays a significant role in many problems. The greatest contribution of Ref. [6] is that it defines the equivalent circuit parameters in terms of the characteristic impedance, rather than *vice versa*, and thereby derives explicit expressions for *C, G, L*, and *R* in terms of the modal fields.

In Sec. 2 of this paper, we present a complete theory of uniform waveguide modes, beginning from first principles. We modify Brews’ definition of the waveguide voltage and current with an alternate normalization devised to simplify the results. We also modify his procedure to simplify the derivation.

In Sec. 3, we proceed to develop a general waveguide circuit theory based on the results of Sec. 2. A number of conclusions presented herein are at odds with not only the electrical circuit theory but also the classical waveguide circuit theory. This is expected, for the classical theory fails to account for losses. The inadequacy of the classical waveguide circuit theory is emphasized by several surprising results of the new theory. For example, the classical theory concludes that the waveguide impedance matrix, like its counterpart in electrical circuit theory, is symmetric when the circuit is composed of reciprocal matter. Here, we demonstrate that this conclusion is not generally valid when lossy waveguide ports are allowed.

Even with the waveguide voltage and current rigorously and consistently defined and with a proper accounting of waveguide loss, another major short-coming of the classical theory remains: the classical waveguide circuit theory fails to appreciate the subtleties of the *scattering matrix*, which, like the impedance matrix, characterizes the circuit, but which relates the traveling wave intensities instead of the waveguide voltages and currents. A good understanding of the scattering matrix, which is related to the impedance matrix by a one-to-one transformation based on the modal characteristic impedance, is vital to a practical waveguide circuit theory, for the scattering matrix is an essential part of an *operational* definition of the impedance matrix. The reason for this, as we discuss in Sec. 4, is that practical waveguide instrumentation is nearly always based on the measurement of waves or similar quantities. In contrast, waveguide voltages and currents, like the fields with which they are defined, are virtually inaccessible experimentally.

The scattering matrix provides a clear distinction between waveguide and electrical circuits, for the scattering matrix has no direct counterpart in electrical circuit theory. Electrical circuits are *not* subject to a traveling wave/scattering matrix description because electrical circuits are not generally composed of uniform waveguides with exponential traveling waves. This is why it is meaningless to speak of the characteristic impedance of an arbitrary electrical port. Nevertheless, the electrical circuit theory mocks the waveguide theory by introducing an arbitrary *reference impedance.* This parameter is used in place of the characteristic impedance in a transformation identical to that relating the corresponding waveguide parameters, resulting in analogous quantities which are often (confusingly) called “traveling waves.” However, since these are not true traveling waves and possess no wave-like characteristics, we prefer to use the term *pseudo-waves*. The relationship between the pseudo-waves is described by a matrix, often (confusingly) called a “scattering matrix,” which we instead call a *pseudo-scattering matrix.*

In contrast to the characteristic impedance, the reference impedance is completely arbitrary. Classical waveguide circuit theory, along with electrical circuit theory, has failed to explicitly recognize this distinction.

While the scattering matrix is incompatible with electrical circuit theory, the pseudo-scattering matrix is compatible with both waveguide and electrical theories. In this paper, we define waveguide pseudo-waves exactly as in the electrical circuit theory, using the waveguide voltage and current and an arbitrary reference impedance. These waveguide pseudo-waves cannot be interpreted as traveling waves but are a linear combination of the traveling waves.

By defining the pseudo-scattering matrix for waveguide as well as electrical circuits, we establish a description common to both. On the other hand, such a common description also exists in the form of the impedance matrix. Why do we require both impedance matrix and pseudo-scattering matrix descriptions? This question has at least three answers, which we now enumerate.

The first answer is that the commonality of the two theories allows the common use of tools developed for one of the two applications. These tools include a number of analytical theorems and results as well as a great deal of measurement and computer-aided design software. Users should be able to take advantage of tools using both impedance matrix and pseudo-scattering matrix descriptions. Furthermore, many tools require *both* descriptions. For example, the Smith chart connects the two in a concise and familiar way.

The second answer has to do with measurement. Electrical circuits are measured in terms of voltages and currents and are therefore fundamentally characterized by impedance matrices. In contrast, the waveguide voltage and current are related to electromagnetic fields which are rarely, if ever, subject to direct measurement. Instead, waveguide circuits are measured in terms of traveling waves and pseudo-waves. For example, a slotted line, traditionally used for waveguide circuit measurement, relies on interference between the traveling waves. Most modern waveguide measurements use a network analyzer. We show in this paper that calibrated network analyzers measure pseudo-waves, defined with respect to a reference impedance determined by the calibration. This reference impedance need not equal the characteristic impedance of the waveguide, so the measured pseudo-waves need not be the actual traveling waves.

The third reason that both impedance and pseudo-scattering descriptions are important is that both are needed to analyze the interconnection of a waveguide with an electrical circuit or with a dissimilar waveguide. Such an analysis typically makes use of two assumptions. The first is that the waveguide fields near the interconnection are composed of a single mode; this assumption may lead to an acceptable result even though the discontinuity virtually always ensures that it is inexact. The second assumption is that the (waveguide or electrical) voltage and current in that single mode are continuous at the interface. This is a generalization of a result from electrical circuit theory that is of questionable validity for waveguide circuits. Due to these two assumptions, any simple analysis of this problem is at best approximate. However, if it is to be applied, the matching conditions on the voltage and current may be directly implemented in terms of impedance parameters, while the waveguides are characterized in terms of scattering or pseudo-scattering parameters. Both sets of parameters are therefore required to solve the problem.

A good example of this kind of problem is the interconnection of a TEM or quasi-TEM waveguide with an electrical circuit which is small compared to a wavelength. In this case, the single-mode approximation may be valid, and the conventional impedance-matching method may be useful if the waveguide voltage and current are defined to be compatible with the electrical voltage and current. The canonical problem of this form is the termination of a planar, quasi-TEM waveguide, such as a microstrip line, with a small, “lumped” resistor. Such problems, while unusual in the study of conventional waveguides, are typical of planar circuits and have become increasingly important with their proliferation. The theory presented here supports the experimental study of these problems using conventional microwave instrumentation.

Although our introduction of pseudo-waves entails some new terminology, these quantities are not new discoveries. They implicitly provide the basis of the conventional “scattering matrix” description of electrical circuit theory. Furthermore, while they have not heretofore been explicitly introduced into waveguide circuit theory, they have been applied, perhaps unconsciously, to waveguide circuits by those unaware of the distinctions between the two theories.

An important contrast to the pseudo-wave theory is an alternative known as the theory of “complex port numbers” [7]. This theory defines what it calls “traveling waves” and corresponding “scattering matrices” in a way that is fundamentally different from that described here. The theory itself was originally applied to electrical circuits and remains popular in that context. It has also been extended to waveguide analysis, where it is known as the theory of “power waves” [8]. Here we demonstrate previously-unknown properties of the “power wave scattering matrix” of a waveguide circuit. Furthermore, we show that the power waves are different from not only the pseudo-waves but also the actual traveling waves propagating in a waveguide. As a result, they present some serious complications, discussed in the text. Practitioners of the waveguide arts must be aware that conventional analysis and measurement techniques do *not* determine relations between power waves. Confusion concerning this matter is prevalent.

In this paper, we comprehensively construct a complete waveguide circuit theory from first principles. Beginning with Maxwell’s equations in an axially independent region, we define the waveguide voltage and current, the characteristic impedance, and the four equivalent circuit parameters of the mode. We then define traveling wave intensities, which are normalized to the characteristic impedance, and pseudo-waves, which are normalized to some arbitrary reference impedance. We discuss in detail the significance of the waves and study expressions for the power. We introduce various matrices relating the voltages, currents, and waves in the ports of a waveguide circuit and describe the properties of those matrices under typical physical conditions. We extensively investigate the problems of measuring these quantities.

Although the normalizations in many of the definitions introduced here are unfamiliar, we have striven to ensure that each parameter is defined in accordance with common usage and with the appropriate units. Awkward definitions are occasionally required to achieve convenient results.

## 2. Theory of a Uniform Waveguide Mode

In this section, we develop a basic description of a waveguide mode. Beginning with Maxwell’s equations, we define the waveguide voltage and current, power, characteristic impedance, and transmission line equivalent circuit parameters. We close with a discussion of the measurement of characteristic impedance.

### 2.1 Modal Electromagnetic Fields

We begin by defining a uniform waveguide very broadly as an axially independent structure which supports electromagnetic waves. In such a geometry, we seek solutions to the source-free Maxwell equations with time dependence *e^+jωt^.* Here we consider only problems involving isotropic permittivity and permeability, although some of the results are easily generalized (see Appendix A). We need to prescribe the appropriate boundary conditions at interfaces and impenetrable surfaces. If the waveguide is transversely open, the region is unbounded, and boundary conditions at infinity, sufficient to ensure finite power, are also required; this excludes leaky modes. The eigenvalue problem is separable and the axial solutions are exponential. In general, there are many linearly independent solutions to this problem, each of which is proportional to a mode of the waveguide. In this paper, we restrict ourselves to consideration of a single mode which propagates in both directions. Most of the results are easily generalized to any finite number of propagating modes.

We introduce complex fields whose magnitude is the root-mean-square of the time-dependent fields, as in Ref. [9], and orient our z-axis along the waveguide axis. For a mode propagating in the forward (increasing z) direction, the normalized modal electric and magnetic fields will be denoted by ***ee****^−γz^* and ***he****^−γz^*, respectively, where ***e*** and ***h*** are independent of z. Although it need not be specified here, some arbitrary but fixed normalization is required to ensure uniqueness of ***e*** and ***h***. The modal propagation constant *γ* is composed of real and imaginary components *α* and *β:*

γ≡α+jβ. (1)

Split ***e*** and ***h*** into their transverse (***e****_t_* and ***h****_t_*) and longitudinal (*e_z_z* and *h_z_z*) components, where *z* is the longitudinal unit vector. As shown in Appendix A, the homogeneous Maxwell equations with isotropic permittivty and permeability can be expanded as

$$\nabla \times e_{t}=-j\omega \mu h_{z}z,$$ (2)

$$\gamma e_{t}+\nabla e_{z}=-j\omega \mu z\times h_{t},$$ (3)

$$\nabla \times h_{t}=+j\omega \epsilon e_{z}z,$$ (4)

$$\gamma h_{t}+\nabla h_{z}=j\omega \epsilon z\times e_{t},$$ (5)

$$\epsilon \nabla \cdot e_{t}+e_{t}\cdot \nabla \epsilon =\epsilon \gamma e_{z},$$ (6)

and

$$\mu \nabla \cdot h_{t}+h_{t}\cdot \nabla \mu =\mu \gamma h_{z}.$$ (7)

We expressly exclude discussion of the case *ω* = 0, to which many of the results in this paper do not apply due to the decoupling of ***e*** and ***h***.

To get a better understanding of the eigenvalue problem, we can eliminate either ***e****_t_* or ***h****_t_* from Eqs. (3) and (5) and thereby derive the explicit expressions for the transverse fields in terms of the axial fields

$$(\omega^{2}\mu \epsilon +\gamma^{2})e_{t}=-\gamma \nabla e_{z}+j\omega \mu z\times \nabla h_{z}$$ (8)

and

$$(\omega^{2}\mu \epsilon +\gamma^{2})h_{t}=-\gamma \nabla h_{z}-j\omega \epsilon z\times \nabla e_{z}.$$ (9)

Differential equations for the axial fields are

$$(\nabla^{2}+\omega^{2}\mu \epsilon +\gamma^{2})e_{z}=\frac{\gamma }{\epsilon }e_{t}\cdot \nabla \epsilon$$ (10)

and

$$(\nabla^{2}+\omega^{2}\mu \epsilon +\gamma^{2})h_{z}=\frac{\gamma }{\mu }h_{t}\cdot \nabla \mu .$$ (11)

These equations are in general quite complicated. In many conventional waveguides, *ϵ* and *μ* are piecewise homogeneous, so the right sides of Eqs. (10) and (11) vanish. Even so, these equations remain complicated since the various fields components are coupled through the boundary conditions.

In general, the solutions of the boundary value problem possess a full suite of field components. In certain cases, it may be possible to find either a TE (*e_z_* = 0) or TM (*h_z_* = 0) solution. Equations (8) and (9) ensure that TEM (*e_z_=h_z_*=0) solutions exist only in domain of homogeneous *μϵ* with the eigenvalue *γ* satisfying *γ^2^ = −ω*^2^*μϵ.* This forbids TEM solutions in the presence of multiple dielectrics, as exist in open planar waveguides or waveguides bounded by lossy conductors.

Equations (2)–(7) prohibit nontrivial modes with either ***e****_t_* = 0 or ***h****_t_* = 0, except when *γ* = 0. This degenerate case, which corresponds to mode of a lossless waveguide operating at exactly the cutoff frequency, is discussed in Appendix C.

### 2.2 Waveguide Voltage and Current

Recall that ***e****_t_, e_z_z*, ***h****_t_*, and *h_z_z*, satisfying Eqs. (2)–(7) with the propagation constant *γ*, represent the fields of the mode propagating in the forward direction. Clearly, the fields ***e****_t_, −e_z_z*, −***h****_t_*, and *h_z_z* satisfy the same equations with a propagation constant of −*γ.* These latter fields represent the normalized *backward* propagating mode. The distinction between the forward and backward modes is made below.

In general, the *total* fields ***E*** and ***H*** in a single mode of the waveguide are linear combinations of the forward and backward mode fields. Their transverse components can therefore be represented by

$$E_{t}=c_{+}e^{-\gamma z}e_{t}+c_{-}e^{+\gamma z}e_{t}\equiv \frac{\upsilon (z)}{\upsilon_{0}}e_{t}$$ (12)

and

$$H_{t}=c_{+}e^{-\gamma z}h_{t}-c_{-}e^{+\gamma z}h_{t}\equiv \frac{i(z)}{i_{0}}h_{t}.$$ (13)

We will call *v* and *i* the *waveguide voltage* and *waveguide current.* The introduction of the normalization constants *v*_0_ and *i*_0_ allows *v* and *v*_0_ to have units of voltage, *i* and *i*_0_ to have units of current, and ***E****_t_*, ***H****_t_*, ***e****_t_*, and ***h****_t_* to have units appropriate to fields. Other waveguide theories omit *v*_0_ and *i*_0_ and therefore require unnatural dimensions.

For basis functions, we have chosen to use the normalized field functions *e_t_*, and ***h****_t_*,, whereas conventional waveguide theories choose arbitrary multiples of ***e****_t_*, and ***h****_t_*. The present formulation is conceptually simpler since ***e****_t_*, and ***h****_t_*, are the fields in the normalized forward-propagating mode. This mode has propagation constant *γ*, waveguide voltage *v*(*z*) *= v*_0_*e^−γz^*, and waveguide current *i*(*z*)*=i*_0_*e^−γz^.* For the normalized backward-propagating mode, the propagation constant is *−γ, v*(*z*) *= v*_0_*e^+γz^*, and *i*(*z*)*= −i*_0_*e^+γz^.*

### 2.3 Power

The net complex power *p*(*z*) crossing a given transverse plane is given by the integral of the Poynting vector^1^ over the cross section *S:*

$$p(z)\equiv \int_{s}E_{t}\times H_{t}*\cdot zdS=\frac{\upsilon (z)i*(z)}{\upsilon_{0}i_{0}^{*}}p_{0},$$ (14)

where we have defined

$$p_{0}\equiv \int_{s}e_{t}\times h_{t}*\cdot zdS.$$ (15)

In accordance with the analogy to electrical circuit theory, we require that

p=υi*. (16)

This cannot be achieved with arbitrary choices of the normalization constants *v*_0_ and *i*_0_. Therefore we impose the constraint

$$p_{0}=\upsilon_{0}i_{0}*,$$ (17)

which allows Eqs. (14) and (16) to be simultaneously satisfied. Either *v*_0_ or *i*_0_ may be chosen arbitrarily; the other is determined by Eq. (17).

The magnitude of *p*_0_ depends on the normalization which determined the modal fields ***e*** and ***h***; in fact, Eq. (15) can even be used to specify the normalization. *The phase of p*_0_ does *not* depend on this normalization since the phase relationship between ***e*** and ***h*** is fixed, to within a sign, by Maxwell’s equations. This sign ambiguity can be resolved by explicitly distinguishing between the forward and backward modes. The most concise means of making this distinction is to define the forward mode as that in which the power flows in the *+z* direction; that is,

Re(p0)⩾0. (18)

The ambiguity remains if Re(*p*_0_) = 0, as occurs in an evanescent waveguide mode. In this case, we use the alternative condition Re(*γ*)>0, which forces the mode to decay with z. With Eq. (18) or its alternative, the phase of *p*_0_ is unambiguous, except in the degenerate case *p*_0_ = 0.

The average power flow *P*(*z*) across 5 is given by the real part of *p*(*z*) as

P(z)≡Re[p(z)]=Re∫sEt×Ht*⋅zdS=Re(υi*). (19)

When only the normalized forward mode is present, the complex power is *p*(*z*) *=p*_0_*e^−^*^2^*^αz^.* When only the normalized backward mode is present, the complex power is *−p*_0_*e^+^*^2^*^αz^.* The associated average powers are Re(*p*_0_)*e^−^*^2^*^αz^* and *−* Re(*p*_0_)e^+2^*^αz^*, respectively. The signs differ because the forward mode carries power in the *+z* direction and the backward mode in the *−z* direction.

The power is *not* generally a linear combination of the forward and backward mode powers, since it is given by the nonlinear expression in Eq. (19). This means that the net real power *P* is in general *not* simply the difference of the powers carried by the forward and backward modes. This issue is discussed at greater length below.

### 2.4 Characteristic Impedance

We define the forward-mode characteristic impedance by

$$Z_{0}\equiv \upsilon_{0}/i_{0}={\left|\upsilon_{0}\right|}^{2}/p_{0}^{*}=p_{0}/{\left|i_{0}\right|}^{2}.$$ (20)

The equivalence of these expressions again demonstrates the analogy to electrical circuit theory. Brews [6,10] also defines the voltage, current, power, and characteristic impedance so as to satisfy Eq. (20) and refers to Schelkunoff s point [11] that the equivalence of these three definitions of *Z*_0_ follows from Eq. (17). The three definitions would in general be inconsistent if *p*_0_, *v*_0_, and *i*_0_ were defined independently (for example, in terms of some power, voltage drop, and current in the waveguide) without regard to Eq. (17).

*Z*_0_ is independent of the normalization of the modal fields ***e*** and ***h*** which affected |p_0_|. While its magnitude *does* depend on the choice of either *v*_0_ or *i*_0_, its *phase* is identical to that of *p*_0_ and therefore independent of all normalizations. As pointed out by Refs. [6] and [10], the phase of the characteristic impedance *Z*_0_ is a fixed, inherent, and unambiguous property of the mode. A sign ambiguity would have remained had we not imposed Eq. (18) since, due to the sign reversal in the current, the characteristic impedance of the backward mode is −*Z*_0_. However, Eqs. (18) and (20) constrain the sign of *Z*_0_ such that

Re(Z0)⩾0. (21)

In particular, as we will see below, the characteristic impedance of any propagating mode of a *lossless* line is real and positive. Equation (21) serves to completely specify *Z*_0_ unless Re(*Z*_0_) = 0, in which case the alternative condition Re(*γ*)>0 suffices to make the distinction.

When only a multiple of the forward-propagating mode exists, then *v*(*z*)/*i*(*z*) =*Z*_0_ for all *z* and at any amplitude. Likewise, when only a multiple of the backward mode exists, then *v*(*z*)/*i*(*z*) = −*Z*_0_. If both forward and backward modes are present, *v*/*i* depends on *z* due to interference between the two.

In order to illustrate the close correspondence between this definition of *Z*_0_ and conventional definitions of the characteristic impedance, we consider the special case of TE, TM, or TEM modes in homogeneous matter. Each of these has fields which satisfy

$$z\times e_{t}=\eta h_{t},$$ (22)

where the wave impedance **η** is constant over the cross section. In this case,

$$Z_{0}=\frac{{\left|\upsilon_{0}\right|}^{2}}{\int_{s}{\left|e_{t}\right|}^{2}dS}\eta .$$ (23)

Since the modal field ***e****_t_* is normalized, the denominator is fixed. The magnitude of *Z*_0_ therefore depends only on *v*_0_. However, *the phase of the characteristic impedance is equal to that of the wave impedance.* This corresponds to most conventional definitions.

For TEM modes, *η* is equal to the intrinsic wave impedance $\sqrt{\mu /\epsilon }$(≈377 Ω in free space), with the result that

$$\arg(Z_{0})=\frac{1}{2}(\arg(\mu )-\arg(\epsilon )).$$ (24)

For example, if *μ* is real then

$$\arg(Z_{0})=-\frac{1}{2}\delta ,$$ (25)

where tan*δ* ≡ Im(*ϵ*)/Re(*ϵ*) is the dielectric loss tangent.

When *v*_0_ is chosen to be the voltage between the ground and signal conductors, *Z*_0_ is equal to the conventional TEM characteristic impedance.

For TE and TM modes,

$$\eta =\sqrt{\frac{\mu }{\epsilon }}{\left(1-\frac{k_{c}^{2}}{\omega^{2}\mu \epsilon }\right)}^{\pm 1/2},$$ (26)

where “ + ” corresponds to TM and “−” to TE and *k*_c_ is the cutoff wavenumber.

### 2.5 Normalization of Waveguide Voltage and Current

Although the phase of either *v*_0_ or *i*_0_ can be chosen arbitrarily, the choice is of little significance. The important quantity is the phase *relationship* between *v*_0_ and *i*_0_, which, due to the constraint (17) and the fact that the phase of *p*_0_ is fixed, is unalterable. The phase relationship between *v*_0_ and *i*_0_ is a unique property of the mode.

The *magnitude* of *Z*_0_ is determined by the choice of *v*_0_ or *i*_0_. Given the constraint [(Eq. 17)], and having selected a modal field normalization, we may independently assign only one of these two variables. One useful normalization defines the constant *v*_0_ by analogy to a voltage using the path integral

$$\upsilon_{0}=-\int_{\mathrm{path}}e_{t}\cdot dl.$$ (27)

The path is confined to a single transverse plane with the restriction that *v*_0_≠0. This can always be arranged unless *e_t_* = 0 everywhere, but this occurs only in the degenerate case *γ* = 0. The integral does not in general represent a potential difference because it depends on the path between a given pair of endpoints. In certain cases, such as when the mode is TM or TEM, the integral depends only on the endpoints, not on the path between them.

Although the path is arbitrary, certain choices are often natural. With a TEM mode, for example, we can put an endpoint on each of two active conductors so that *v*_0_ becomes the path-independent voltage drop across them at *z* = 0 in the normalized mode. In this case, *Z*_0_ is equal to the conventional TEM characteristic impedance. We may not have both endpoints on the same conductor, for then *v*_0_ = 0. The same is true of TM modes.

A result of Eq. (27) is that *v* is also analogous to voltage:

$$\upsilon (z)=-\int_{\mathrm{path}}E_{t}(z)\cdot dl.$$ (28)

The normalization in Eq. (27) yields what is known as a “power-voltage” definition of the characteristic impedance, even though the “voltage” is not an actual potential difference. Another useful possibility is a “power-current” definition, choosing *i*_0_ to be a current. Yet another choice, popular for hollow waveguides, is to normalize so that |*Z*_0_| = l. It is not our intent to debate the issue of the optimal definition. However, it is only the magnitude, not the phase, of *Z*_0_ that is open for discussion.

A “voltage-current” definition, popular in the literature, is generally forbidden by Eq. (20), since an arbitrarily specified *v*_0_ and *i*_0_ may not be of the appropriate phase to satisfy *v*_0_*/i*_0_*=Z*_0_.

Appendix F includes a table displaying the effects of renormalizing *v*_0_ and *e*_0_ on all of the parameters used in this work.

### 2.6 Transmission Line Equivalent Circuit

We now develop a transmission line analogy by defining real equivalent circuit parameters *C, L, G*, and *R*, analogous to the capacitance, inductance, conductance, and resistance per unit length of conventional transmission line theory. The four parameters are defined by

$$j\omega C+G\equiv \frac{\gamma }{Z_{0}}$$ (29)

and

$$j\omega L+R\equiv \gamma Z_{0}.$$ (30)

**Fig. 1:**
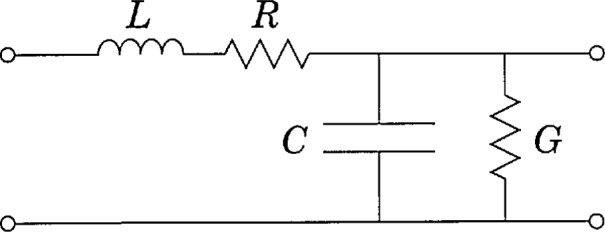
Equivalent circuit model of transmission line.

Equations (29) and (30) are identical to those derived from the electrical circuit theory description of a transmission line with distributed shunt admittance *jωC + G* and series impedance *jωL + R*, as shown in Fig. 1. These quantities also appear in the conventional transmission line equations satisfied by *v* and *i*:

$$\frac{d\upsilon }{dz}=-(j\omega L+R)i$$ (31)

and

$$\frac{di}{dz}=-(j\omega C+G)\upsilon .$$ (32)

Although Eqs. (29) and (30) provide unique definitions of the four circuit parameters, it is possible to cast them into another form which is more convenient for many purposes, as is done by Brews [6]. A simpler derivation, given in Appendix B, shows that the circuit parameters are given exactly by

$$C=\frac{1}{{\left|\upsilon_{0}\right|}^{2}}\left[\int_{s}\epsilon^{\prime }{\left|e_{t}\right|}^{2}dS-\int_{s}\mu^{\prime }{\left|h_{z}\right|}^{2}dS\right],$$ (33)

$$L=\frac{1}{{\left|i_{0}\right|}^{2}}\left[\int_{s}\mu^{\prime }{\left|h_{t}\right|}^{2}dS-\int_{s}\epsilon^{\prime }{\left|e_{z}\right|}^{2}dS\right],$$ (34)

$$G=\frac{\omega }{{\left|\upsilon_{0}\right|}^{2}}\left[\int_{s}\epsilon^{\prime \prime }{\left|e_{t}\right|}^{2}dS+\int_{s}\mu^{\prime \prime }{\left|h_{z}\right|}^{2}dS\right],$$ (35)

and

$$R=\frac{\omega }{{\left|i_{0}\right|}^{2}}\left[\int_{s}\mu^{\prime \prime }{\left|h_{t}\right|}^{2}dS+\int_{s}\epsilon^{\prime \prime }{\left|e_{z}\right|}^{2}dS\right].$$ (36)

Here *ϵ ≡ ϵ′–jϵ″* and *μ≡μ′ – μ″.* In passive media, the four real components *ϵ′*, *ϵ″*, *μ′*, and *μ″* are all nonnegative. Metal conductivity is not included as an explicit term in *ϵ* but is instead absorbed in *ϵ″*. In general, of course, *ϵ* and *μ*. depend on *ω.*

The parameters *C, L, G*, and *R* depend on the same normalization that determines the magnitude of *Z*_0_. For instance, when *i*_0_ is chosen to be the voltage between two active conductors in a lossless TEM line, then *C* and *L* are the conventional capacitance and inductance per unit length. Certain combinations of these parameters, notably *G*/(*ωC*), *R*/(*ωL*), *RC, RG, LC*, and *LG*, are normalization-independent. For example, *LC = ϵ′μ′* for a TEM line.

Equations (33) through (36) have many applications. In addition to providing a means of numerically calculating the circuit parameters from known fields, they offer opportunities for analytical calculations and approximations as well. The quadratic form in which the fields appear make them particularly useful for these purposes. Another major role they serve is in the attribution of circuit-parameter components to portions of the cross section. For example, it is common to divide the inductance *L* into an “external” inductance in the dielectric and an “internal” inductance in the imperfect metal. Such a division cannot be undertaken using only Eq. (30) but is readily obtainable by dividing the surface integral in Eq. (34) into dielectric and metal regimes.

Equations (29) and (30) imply the familiar expressions

$$\gamma =\sqrt{\left(j\omega L+R)(j\omega C+G\right)}$$ (37)

and

$$Z_{0}=\sqrt{\left(j\omega L+R)/(j\omega C+G\right)}.$$ (38)

The pairs of roots in Eqs. (37) and (38) correspond to the presence of both forward and backward modes, each of which have identical *C, L, G*, and *R* but opposite *γ* and *Z*_0_. To distinguish the two, recall from Eq. (21) that the forward mode is defined such that Re(*Z*_0_)≥0. Either Eq. (29) or (30) can then be used to distinguish between the two values of *γ*. If the waveguide material is passive, then Eqs. (35) and (36) ensure that *G* and *R* are both nonnegative, which requires that *α* = Re(*γ*) ≥ 0. Thus, the fields of the mode that we have defined as the forward one must decay with increasing *z* in a lossy system. In general, however, the sign of *α* does not distinguish the forward and backward modes since *α* = 0 in energy-conserving modes and may be negative in the presence of active media. Nevertheless, Eq. (18) ensures that the forward mode carries power only in the *+z* direction.

*C* and *L* are typically positive for modes of common interest, in which the energy is primarily carried in the transverse fields and the second integrals of Eqs. (33) and (34) are relatively small. On the other hand, *C* and *L* may be zero or negative in certain cases. For instance, in the lossless case in which *ϵ*″ = *μ*″ =0, *G =R* =0 and Eqs. (37) and (38) become

$$(\epsilon^{\prime \prime }=\mu^{\prime \prime }=0)\Rightarrow \gamma =j\omega \sqrt{LC}$$ (39)

and

$$(\epsilon^{\prime \prime }=\mu^{\prime \prime }=0)\Rightarrow Z_{0}=\sqrt{\frac{L}{C}}$$ (40)

As shown in Appendix C, the modes of a *lossless* waveguide, except those with *p*_0_=0, either propagate without attenuation (*α* = Re(*γ*) = 0) or are evanescent (*α* > 0 but *β* = Im(*γ*) = 0). For the propagating modes, therefore, *LC* is nonnegative and thus *Z*_0_ and *p*_0_ are real. For the evanescent modes, *Z*_0_ and *p*_0_ are imaginary and the mode carries no average real power. Equation (39) shows that, for evanescent modes, either *L* or C, but not both, must be negative. For instance, TM modes have *h_z_* = 0, so that C cannot be negative. As a result, *L* > 0 for propagating TM modes and *L* < 0 for evanescent TM modes. Complementary statements hold for lossless TE modes.

In lossy waveguides, we can no longer strictly distinguish “propagating” from “evanescent” modes, since generally *α* and *β* are both nonzero. Therefore, if we perturb a lossless TM mode by the addition of a minuscule amount of *ϵ″*, we find a mode that is not evanescent in a strict sense (since *β ≠* 0) but nevertheless has *L* < 0. In this way we prove that not all modes with *L* < 0 or C < 0 are strictly evanescent.

The allowed range of the phases of *γ* and *Z*_0_ is determined by Eqs. (37) and (38). We assume for the moment that *G* and *R* are nonnegative, as in passive structures. In this case, if *C* and *L* are positive, then *γ* lies in the first quadrant and −45°≤ arg(*Z*_0_) ≤ 45°. If in addition *G* = 0, a good approximation in many common quasi-TEM waveguides, then 45° ≤ arg(*γ*) ≤ 90° and −45° ≤ arg(*Z*_0_) ≤ 0°. If instead *R*=0, then again 45° ≤ arg(*γ*) ≤ 90°, but now 0° ≤ arg(*Z*_0_) ≤ 45°. In lossless propagating modes, *γ* is positive imaginary and *Z*_0_ positive real. *Z*_0_ is also real in lossy lines in the special case *G*/(*ωC*)*=R*/(*ωL*).

**Fig. 2:**
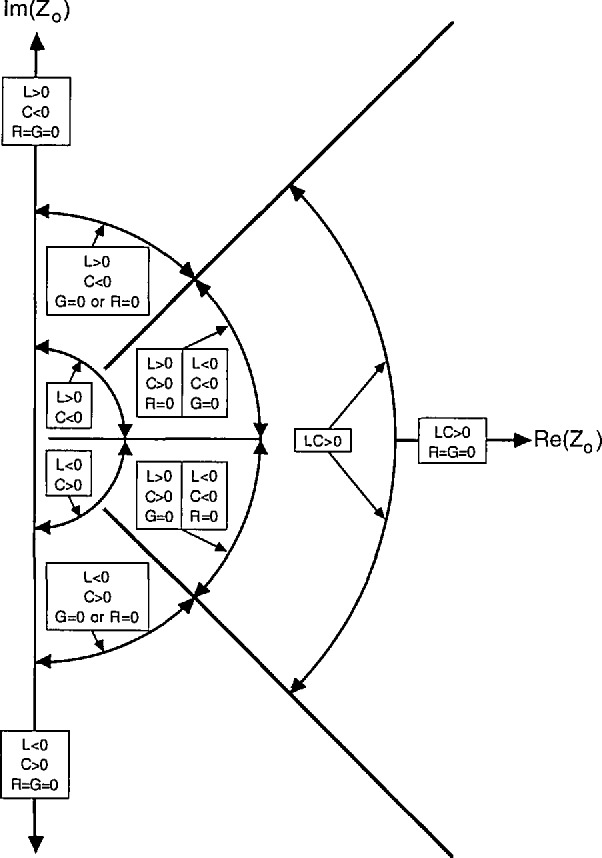
Allowed ranges of the phase of *Z*_0_ for various signs of the equivalent circuit parameters. The figure gives no indication of the magnitude of *Z*_0_. *G* and *R* are assumed to be nonnegative.

**Fig. 3:**
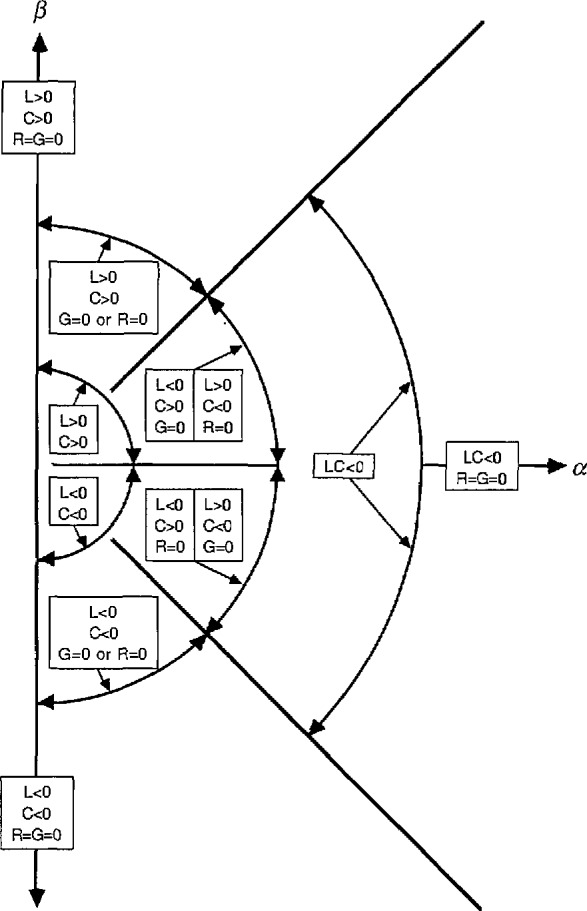
Allowed ranges of the phase of *γ* for various signs of the equivalent circuit parameters. The figure gives no indication of the magnitude of *γ*. *G* and *R* are assumed to be nonnegative.

Figures (2) and (3) illustrate the allowed range of the phase of *Z*_0_ and *γ* for various cases, as distinguished by the signs of *L* and *C. G* and *R* are assumed nonnegative in these figures.

Let us compare the current results to the conventional theory of TEM lines. For a lossless TEM line, *G* and *R* vanish, as do the second integrals in C and *L*. The remaining integrals in *C* and *L* are simply the energy per unit length stored in the electric and magnetic fields, respectively. Thus the expressions for *C* and *L* are simply the conventional expressions for the dc capacitance and inductance per unit length, as given by Collin [3]. When the dielectric is lossy but *μ″* is zero, the mode may remain TEM but a shunt conductance *G*, given by the first term of Eq. (35) as in Ref. [3], is present.

For a general TEM line,

$$(\mathrm{TEM}):Z_{0}^{2}=\frac{\epsilon \prime \mu }{\epsilon \mu \prime }\frac{L}{C}=\frac{\mu }{\mu \prime^{2}\epsilon }L^{2}=\frac{\mu \epsilon \prime^{2}}{\epsilon }\frac{1}{C^{2}},$$ (41)

which takes a more familiar form when *ϵ*″ = *μ″* = 0.

When the metal boundaries are lossy or the dielectric is inhomogeneous, the mode is non-TEM. The second integrals in *C* and *L*, which are absent in Ref. [3], are quadratic in the longitudinal fields and may, in some quasi-TEM cases, prove to be negligible compared to the first terms. The expressions for *C* and *G* in general include contributions due to fields inside the metal that are not often appreciated. A nonzero series resistance *R*, given by the second integral in Eq. (36), may also appear whenever *e_z_* and *ϵ″* are nonzero; the integral extends over a lossy dielectric as well as an imperfect conductor. Collin does not provide a surface-integral expression for *R*, but it can be shown that Eq. (36) reduces to Collins line-integral expression when the surface-impedance approximation is invoked and the dielectric is lossless.

### 2.7 Effective Permittivity and the Measurement of Characteristic Impedance

It is useful and customary to define the effective relative dielectric constant (or permittivity) by

$$\epsilon_{r,\mathrm{eff}}\equiv -{\left(c\gamma /\omega \right)}^{2}.$$ (42)

where *c* is the speed of light in vacuum. This definition equates *γ* to the propagation constant of a TEM mode in a fictitious medium of permittivity *ϵ*_r,eff_ *ϵ*_0_ and permeability *μ*_0_. We have no need to define an effective permeability.

Using Eq. (37),

$$\epsilon_{r,\mathrm{eff}}=\frac{c^{2}}{\omega^{2}}\left[\omega^{2}LC-RG-j\omega \left(LG+RC\right)\right].$$ (43)

If, as is most common, *C,L,G*, and *R* are nonnegative, then Im(*ϵ*_r,eff_)⩽0. Although Re(*ϵ*_r,eff_) is typically positive, it becomes negative in lossy lines at low frequencies if *RG >ω*^2^*LC.* It is also negative for lossless, evanescent modes.

An alternative form of Eq. (29) is

$$Z_{0}=\frac{\sqrt{\epsilon_{r,\mathrm{eff}}}}{cC\left(1+G/j\omega C\right)},$$ (44)

which, as discussed in Ref. [12], may be applicable to the determination of *Z*_0_. For example, if G/(*ωC*) is known, the phase of *Z*_0_ is determined by the phase of *ϵ*_r,eff_. For TM modes in homogeneous dielectric, G/(*ωC*) = tan*δ*, which is typically much less than 1 and can often be neglected. The same is true for typical quasi-TEM modes. In these cases, *C* is nearly independent of frequency and may be readily determinable [13]. If so, then Eq. (44) provides the magnitude as well as the phase of *Z*_0_. This provides a practical method of determining *Z*_0_, since *ϵ*_r,eff_ may be readily measured using standard microwave instrumentation to measure *γ.* By contrast, a direct measurement of *Z*_0_ is impractical. For instance, the phase of *Z*_0_ is defined as the phase of the complex power *p*_0_, a quantity which is difficult to assess directly without detailed knowledge of the modal fields.

A similar method of determining *Z*_0_ makes use of the relationship between *Z*_0_, *γ, L*, and nductance and resistive loss typically make *R*/(*ωL*) nonnegligible and *L* and *R* strongly dependent on resistivity and frequency. In other cases, however, it may prove useful.

## 3. Waveguide Circuit Theory

In this section, we apply the results of Sec. 2 to develop a waveguide circuit theory. We first discuss traveling waves and pseudo-waves for a single uniform waveguide. These form the basis of the scattering and pseudo-scattering matrices. We also introduce the cascade and impedance matrices and discuss the transformation of reference impedance, concluding with an investigation of the load impedance.

### 3.1 Traveling Wave Intensities

We define the forward and backward traveling wave intensities (or simply traveling waves) *a*_0_ and *b*_0_ by normalizing the forward and backward modes of Eqs. (12) and (13):

a0≡Re(p0)c+e−γz=Re(p0)2υ0(υ+iZ0) (45)

and

b0≡Re(p0)c−e+γz=Re(p0)2υ0(υ−iZ0), (46)

where the positive square root is mandated. This power normalization ensures that, in the absence of the backward wave, the unit forward wave with *a*_0_ = 1 carries unit power.

It can be shown that *a*_0_ and *b*_0_ are independent of the arbitrary normalization of *v*_0_. While their phases depend on the phase of the modal field *e_t_* in the same way that c+ and c− do, *a*_0_ and *b*_0_ are independent of the magnitude of *e_t_.* This normalization-independence suggests that *a*_0_ and *b*_0_ are physical waves rather than simply mathematical artifacts.

Assuming that Re(*Z*_0_)≠0, Eqs. (45) and (46) imply

υ(z)=υ0Re(p0)(a0+b0) (47)

and

i(z)=i0Re(p0)(a0−b0). (48)

From Eq. (19), the real power is therefore

P(z)=|a0|2−|b0|2+2Im(a0b0*)Im(Z0)Re(Z0). (49)

This demonstrates that the net real power *P* crossing a reference plane is *not* equal to the difference of the powers carried by the forward and backward waves acting independently, except when the characteristic impedance is real or when either *a*_0_ or *b*_0_ vanishes.

Although Eq. (49) is awkward and somewhat counterintuitive, it is not an artifact of the formulation but an expression of fundamental physics. Normalizations do not play a role, for the result is independent of the normalizations of *e_t_* and *v*_0_. Only the phase of *Z*_0_ appears and, as we have seen, this phase is not arbitrary.

In the evanescent case, Re(*p*_0_) = Re(*Z*_0_) = 0, so that neither the forward nor backward wave individually carries real power. In this case, Eq. (49) is indeterminate. To resolve the problem, we can express Eq. (49) in the form

P(z)=|a0|2−|b0|2+2Im(p0)Im(c+c−*), (50)

since *β* = 0 for evanescent waves. When Re(*p*_0_) = 0, both *a*_0_ and *b*_0_ vanish as a result of the power normalization of Eqs. (45) and (46), but the last term-may be nonzero. This means, that, although the forward and backward cutoff waves each carry no real power, power may be transferred if both waves exist. Thus, as we expect, power *may* traverse a finite length of lossless waveguide in which all modes are strictly cut off. This familiar case exemplifies the fact that the net power may fail to equal the sum of the individual wave powers.

The reflection coefficient *Γ*_0_ is defined by

$$\Gamma_{0}\left(z\right)\equiv \frac{b_{0}\left(z\right)}{a_{0}\left(z\right)}.$$ (51)

The power can be expressed in terms of *Γ*_0_ by

P=|a0|2[1−|Γ0|2−2Im(Γ0)Im(Z0)Re(Z0)], (52)

which is similar to a result on p. 27 of Ref. [2]. As noted in Ref. [2], |*Γ*_0_|^2^ is not a power reflection coefficient and may exceed 1 if *Z*_0_ is not real.

### 3.2 Pseudo-Waves

We now introduce another set of parameters, the pseudo-waves, which, in contrast to the traveling waves, *are* mathematical artifacts but may have convenient properties. We first introduce an arbitrary *reference* impedance *Z*_ref_, with the sole stipulation Re(*Z*_ref_)⩾0. We then define the complex *pseudo-wave amplitudes* (or simply *pseudo-waves) a* and *b* by

a(Zref)≡[|υ0|υ0Re(Zref)2|Zref|](υ+iZref) (53)

and

b(Zref)≡[|υ0|υ0Re(Zref)2|Zref|](υ−iZref). (54)

Although *a* and *b* depend on *z* (through *v* and *i*), we have chosen not to explicitly list *z* as an argument but instead to concentrate on the parameter *Z*_ref_, which plays a more important role in the remainder of this development.

The inverse relationships to Eqs. (53) and (54) are

υ=[υ0|υ0||Zref|Re(Zref)](a+b) (55)

and

i=1Zref[υ0|υ0||Zref|Re(Zref)](a−b). (56)

Positive square roots are again mandated in Eqs. (53) through (56).

With these definitions, Eq. (19) becomes

P=|a|2−|b|2+2Im(ab*)Im(Zref)Re(Zref). (57)

*P, v*, and *i* were defined earlier and do not depend on *Z*_ref_.

The pseudo-reflection coefficient *Γ*, defined by

$$\Gamma \left(Z_{\mathrm{ref}}\right)\equiv \frac{b\left(Z_{\mathrm{ref}}\right)}{a\left(Z_{\mathrm{ref}}\right)},$$ (58)

depends on *Z*_ref_. The analog of Eq. (52) is

P=|a|2[1−|Γ|2−2Im(Γ)Im(Zref)Re(Zref)]. (59)

Comparing Eqs. (45) and (46) with Eqs. (53) and (54), we see that *a*(*Z*_0_) = *a*_0_ and *b*(*Z*_0_) = *b*_0_. Although the multiplicative factor in Eqs. (53) and (54) is complicated, it is the only factor that satisfies this criterion and also ensures that *a* and *b* satisfy the simple power expression Eq. (57).

Since the pseudo-waves are equivalent to the actual traveling waves when the reference impedance is equal to the characteristic impedance of the mode, this is the natural choice of reference impedance. On the other hand, it is not always the most convenient choice. For instance, when *Z*_0_ varies greatly with frequency, as is often the case in lossy lines [12], the resulting measurements using *Z*_ref_=*Z*_0_ may be difficult to interpret; a constant *Z*_ref_ may be preferable. Furthermore, the characteristic impedance of a given mode is often unknown and difficult to measure. In such cases, the fact that *Z*_ref_=*Z*_0_ does not suffice to provide a numerical value for *Z*_ref_, which is required in order to make use of Eqs. (55) through (57).

Other choices of reference impedance are also well motivated. In particular, if *Z*_ref_ is chosen to be *real*, the crossterm in Eq. (57) disappears. The result is the conventional expression in which the power is simply the difference of la |*a*|^2^ and |*b*|^2^. The choice of real *Z*_ref_ therefore simplifies subsequent calculations and allows the application of a number of standard results which arise from the conventional expression. For example, conservation of energy ensures that the net power *P* into a passive load is nonnegative. If *Z*_ref_ is real, Eq. (59) implies that the load’s reflection coefficient has magnitude less than 1; that is, it “stays inside the Smith chart.” This need not be true for complex *Z*_ref_. Another example is the conventional result that the maximum power available from a generator is that power which would be delivered to a load whose reflection coefficient is the complex conjugate of the generators reflection coefficient. In the general case, this result applies only to pseudo-reflection coefficients using a real reference impedance.

One more choice of reference impedance is in common use: that which makes *b*(*Z*_ref_) vanish at a given point on the line. Such a choice (*Z*_ref_*=v/i*) also simplifies Eq. (57), although only at the particular *z* and for a particular termination. The primary effect of this choice of *Z*_ref_ is to make the pseudo-reflection coefficient vanish. As discussed later in this paper, many calibration schemes force the pseudo-reflection coefficient of some “standard” termination, usually a resistive load, to vanish. Those schemes thereby implicitly impose this particular choice of reference impedance.

Unfortunately, the quantities *a* and *b* are proportional to the forward and backward traveling waves *only* if *Z*_ref_= *Z*_0_; otherwise, the pseudo-waves are linear combinations of the forward and backward waves. For example, suppose that we have an infinite waveguide with all sources in *z* > 0. For *z* < 0, we know that *a*_0_ = 0; no wave is incident from this side. However, unless *Z*_ref_= *Z*_0_, we will find that *a* and *b* are both nonzero in this case.

**Fig. 4:**
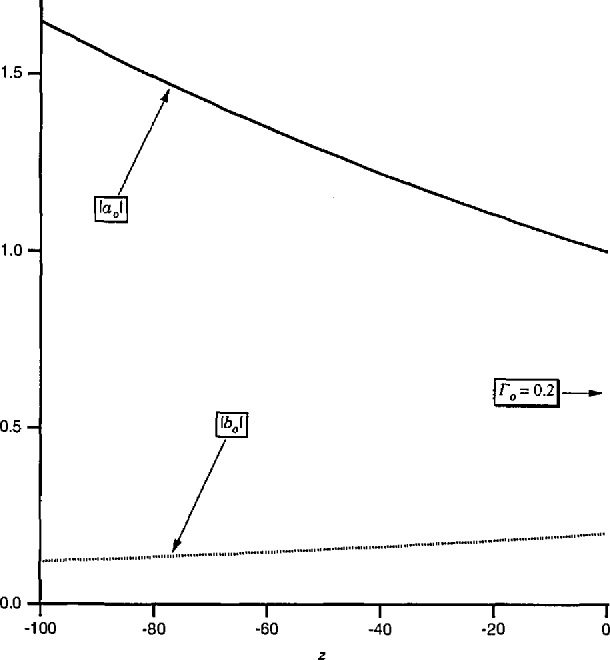
The magnitudes of the incident (*a*_0_) and reflected (*b*_0_) traveling waves near a termination at *z* =0 with reflection coefficient *Γ*_0_ = 0.2. The propagation constant is 0.005+0.1*j*. The waves depend exponentially on *z.*

**Fig. 5:**
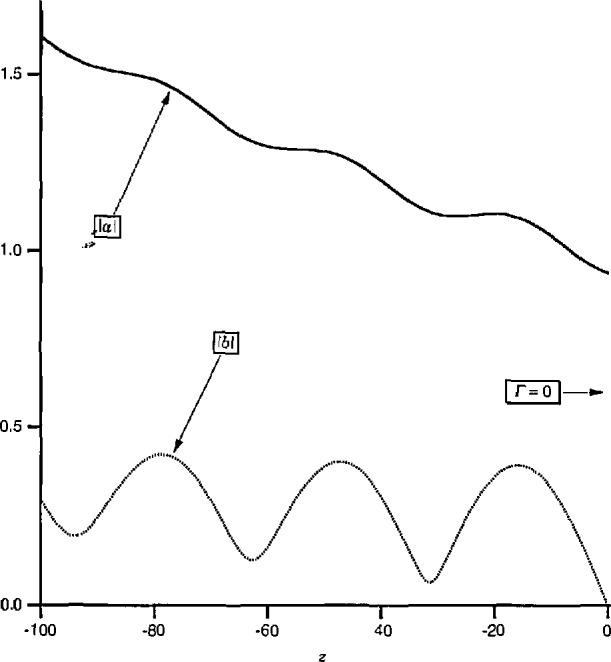
The magnitudes of the pseudo-waves *a* and *b* for the example of Fig. 4. The reference impedance *Z*_ref_ is chosen so as to make the pseudo-reflection coefficient *Γ*(*Z*_ref_) vanish at the termination reference plane. Since the waves depend in a complicated fashion on *z*, *Γ*(*Z*_ref_) vanishes *only* at *z* = 0.

Another contrast is that, as a function of *z, a*_0_ and *b*_0_ have a simple exponential dependence while *a* and *b* are complicated functions of *z* due to interference between the forward and backward traveling waves. For illustration, Fig. 4 plots the magnitudes of *a*_0_ and *b*_0_ for a line which is uniform in *z* < 0 but has an obstacle of reflection coefficient *Γ*=0.2 located at *z* = 0. In contrast, Fig. 5 plots the magnitudes of the associated pseudo-waves *a* and *b* with *Z*_ref_ chosen to make *b* vanish at *z* = 0. Figure 5 demonstrates not only the complicated behavior of *a* and *b* with respect to *z* but also the fact that the change of reference impedance forces *b* to vanish at only a *single* point. It is clearly unrealistic to interpret *a* and *b* as *“incident”* and “reflected” waves.

In contrast to *a*_0_ and *b*_0_, *a* and *b* generally depend on the normalization which determines |*v*_0_|, |*i*_0_| nd |*Z*_0_|. This dependence helps to explain a potential paradox. Assume, for instance, that *Z*_0_=50 Ω. If *Z*_ref_=50 Ω, then the pseudo-waves are equal to the traveling waves. Now, since |*Z*_0_| is arbitrary, depending on how we define *v*_0_, we can easily refine *Z*_0_ to, say, 100 Ω. Are not the pseudo-waves still equal to the traveling waves, even though *Z*_ref_*≠Z*_0_? In fact, they are not, for the change in *v*_0_ leads to a renormalization of *v* and *i* [see Eqs. (12) and (13)] and therefore a renormalization of *a* and *b* through Eqs. (53) and (54). Thus, the pseudo-waves are no longer equal to the traveling waves unless we shift *Z*_ref_ to 100 Ω as well. This normalization dependence of the pseudo-waves, in contrast to the traveling waves, further illustrates the fact that they are not physical waves but instead only mathematical artifacts.

Finally, the condition Re(*Z*_ref_)⩾0 that we have imposed on the reference impedance corresponds to the condition Re(*Z*_0_)⩾0 that we imposed earlier on the characteristic impedance. Therefore, it is always possible to choose *Z*_ref_ *= Z*_0_.

Since the most convenient choice of *Z*_ref_ depends on the application, it will prove useful to construct a procedure to transform the pseudo-waves in accordance with a change of reference impedance. This is considered below.

### 3.3 Voltage Standing Wave Ratio

To illustrate the distinction between the traveling waves and the pseudo-waves, we introduce the voltage standing wave ratio (VSWR). For simplicity, we limit discussion to the lossless case *a* = 0, in which case the fields in the waveguide are strictly periodic in *z* with period 2π/*β*. The VSWR is defined to be the ratio of the maximum to the minimum electric field magnitude, which reduces to

$$\begin{array}{l} \mathrm{VSWR}\equiv \frac{\underset{z}{\max}\left|E_{t}\left(z\right)\right|}{\underset{z}{\min}\left|E_{t}\left(z\right)\right|}=\frac{\underset{z}{\max}\left|\upsilon \left(z\right)\right|}{\underset{z}{\min}\left|\upsilon \left(z\right)\right|}\\ \;\;=\frac{\left|a_{0}\right|+\left|b_{0}\right|}{\left|a_{0}\right|-\left|b_{0}\right|}=\frac{1+\left|\Gamma_{0}\right|}{1-\left|\Gamma_{0}\right|}. \end{array}$$ (60)

In the lossless case, the magnitudes of *a*_0_, *b*_0_, and *Γ*_0_ are independent of *z.*

Equation (60) illustrates that the VSWR, a quantity which is determined solely from the electric fields, is directly related to the ratio of traveling waves. In fact, it is the interference between these traveling waves that produces the periodicity. The pseudo-waves cannot be measured by such a procedure because they have no physical manifestation.

The pseudo-waves reduce to the traveling waves when the reference impedance is equal to the characteristic impedance. Therefore, the reference impedance of the reflection coefficient derived from a VSWR measurement is equal to *Z*_0_. This provides another argument that *Z*_0_ is the natural choice of reference impedance.

### 3.4 Scattering and Pseudo-Scattering Matrices

Consider a linear waveguide circuit which connects an arbitrary number of (generally) nonidentical, uniform semi-infinite waveguides which are uncoupled away from the junction. In each waveguide, a cross-sectional reference plane is chosen at which only a single mode exists. If the mode of interest is dominant, this can be ensured by choosing the reference plane sufficiently far from the junction that higher-order modes have decayed to insignificance.

For each waveguide port *i*, we choose a reference impedance $Z_{\mathrm{ref}}^{i}$, in terms of which the pseudo-wave amplitudes $a_{i}\left(Z_{\mathrm{ref}}^{i}\right)$ and $b_{i}\left(Z_{\mathrm{ref}}^{i}\right)$ at port *i* are defined by Eqs. (53) and (54). The orientation is such that the “forward” direction is *toward* the junction. We define column vectors a and b whose elements are the *a*_i_, and *b*_i_. The vector of outgoing pseudo-waves b is linearly related to the vector of incoming pseudo-waves a by the pseudo-scattering matrix S:

b=Sa. (61)

Although S depends on the choice of reference impedance at each port, we have suppressed notation which would explicitly acknowledge that fact. We likewise define the vectors of incoming and outgoing traveling wave intensities a_0_ and b_0_ whose elements are the *a*_0_ and *b*_0_. These two vectors are related by the (true) scattering matrix S^0^:

$$b_{0}=S^{0}\;a_{0}.$$ (62)

If $Z_{\mathrm{ref}}^{i}={Z_{0}}^{i}$ for each port *i*, then S = S^0^. In other words, the pseudo-scattering matrix is equal to the scattering matrix when the reference impedance at each port is equal to the respective characteristic impedance.

The reflection coefficient *Γ*_0_ is the single element of the scattering matrix S of a one-port. The same is also true of *Γ* and S.

We can say more about S in special cases. For example, the net power into a *passive* circuit is non-negative. From (57), this requires that

Re(a†[I−S†S+2jVS]a)⩾0. (63)

where “†” indicates the Hermitian adjoint (conjugate transpose) and V is a diagonal matrix with elements equal to Im(Zrefi)/Re(Zrefi). If the circuit is lossless, the inequality in Eq. (63) can be replaced by an equality. If all of the reference impedances are real, then Eq. (63) implies that I – S^†^S is positive semi-definite. If, in addition, the circuit is lossless, then S^†^S = I; that is, S is unitary.

Another useful property of S is a result of electromagnetic reciprocity and is therefore demonstrable when all the materials comprising the junction have symmetric permittivity and permeability tensors; in using Eqs. (2)–(7), we have already assumed as much in the waveguides themselves. As shown in Appendix D and also in Ref. [14], the reciprocity condition is

SjiSij=KiKj1−jIm(Zrefi)/Re(Zrefi)1−jIm(Zrefj)/Re(Zrefj), (64)

where the reciprocity factor *K_i_* is given by

$$K_{i}\equiv \frac{{\tilde{p}}_{0i}}{p0i*}.$$ (65)

Here

$${\tilde{p}}_{0}\equiv \int_{s}e_{t}\times h_{i}\cdot z\;dS$$ (66)

and the additional subscript *i* refers to the port. When *Z*_ref_ = *Z*_0_ at each port, Eq. (64) simplifies to

Sji0Sji0=p∼0iRe(p0i)Re(p0j)p∼0j. (67)

The significance of Eq. (64) is that, in contrast to conventional expectations, electromagnetic reciprocity does not necessarily lead to symmetry of the S matrix. In lossless waveguides, *K_i_* = 1 and *Z*_0_ is real, so S^0^ is symmetric and we need only choose each reference impedance equal to the corresponding characteristic impedance to ensure a symmetric S. In lossy waveguides, *K*_i_ is not generally equal to 1. Although *K_i_*≈1 for typical waveguides, calculations show that it may be much less than 1 in certain guides with very lossy dielectrics [14]. Furthermore, it is not always desirable or even possible to choose a real reference impedance, and a complex reference impedance generally destroys the symmetry of S even when *K_i_* = 1. For devices with more than two ports, it is not generally possible to choose the reference impedances so as to make S symmetric. S can always be made symmetric for a two-port, but the phase of the appropriate *Z*_ref_ at each port depends on *K_i_* at both ports.

Experiments which illustrate the effect of the phase of the reference impedance on the symmetry of S are reported in Refs. [14] and [15].

### 3.5 The Cascade Matrix

Equation (61) denotes a linear relation between the *a_i_* and *b_i_*. If the circuit of interest is a two-port with *S*_21_≠0, we can express the same relationship using the cascade matrix R, which relates the various pseudo-waves by

$$\left[\begin{array}{c} b_{1}\left(Z_{\mathrm{ref}}^{i}\right)\\ a_{1}\left(Z_{\mathrm{ref}}^{i}\right) \end{array}\right]=R^{ij}\left[\begin{array}{c} a_{2}\left(Z_{\mathrm{ref}}^{j}\right)\\ b_{2}\left(Z_{\mathrm{ref}}^{j}\right) \end{array}\right].$$ (68)

The indices in the superscript of R*^ij^* indicate that the reference impedance at port 1 is $Z_{\mathrm{ref}}^{i}$ and that at port 2 is $Z_{\mathrm{ref}}^{j}$.

Formulas for the conversion between scattering and cascade matrices are readily available [4,16]. For completeness, we repeat them here:

$$R=\frac{1}{S_{21}}\left[\begin{array}{cc} S_{12}S_{21}-S_{11}S_{22} & S_{11}\\ -S_{22} & 1 \end{array}\right]$$ (69)

and

$$S=\frac{1}{R_{22}}\left[\begin{array}{cc} R_{12} & R_{11}R_{22}-R_{12}R_{21}\\ 1 & -R_{21} \end{array}\right].$$ (70)

The cascade matrix of two series-connected two-ports is the product of the two cascade matrices as long as the connecting ports are composed of identical waveguides, with identical reference impedances, joined without discontinuity. Since this holds true regardless of the reference impedances, the introduction of terminology such as “pseudo-cascade matrix” would be needlessly confusing. We will, however, introduce the special notation R^0^ to describe the cascade matrix which satisfies

$$\left[\begin{array}{c} b_{01}\\ a_{01} \end{array}\right]=R^{0}\left[\begin{array}{c} a_{02}\\ b_{02} \end{array}\right].$$ (71)

R is equal to R^0^ when $Z_{\mathrm{ref}}^{i}={Z_{0}}^{i}$ for each port *i*.

### 3.6 The Impedance Matrix

The impedance matrix *Z* relates the column vectors v and i, whose elements are the waveguide voltages and currents at the various ports:

v=Zi. (72)

In contrast to S and R, *Z* is independent of the reference impedance since v and i are also. This makes *Z* particularly interesting for metrological purposes. *Z* does, however, depend on the normalization of *v*_0_.

The relation between S and *Z* is explored in Appendix E. The results are

$$\begin{array}{l} S=U\left(Z-Z_{\mathrm{ref}}\right){\left(Z+Z_{\mathrm{ref}}\right)}^{-1}U^{-1}=\\ U\left({\mathrm{ZZ}}_{\mathrm{ref}}^{-1}-I\right){\left({\mathrm{ZZ}}_{\mathrm{ref}}^{-1}+I\right)}^{-1}U^{-1} \end{array}$$ (73)

and inversely

$$Z={\left(I-U^{-1}\mathrm{SU}\right)}^{-1}\left(I+U^{-1}\mathrm{SU}\right)Z_{\mathrm{ref}}.$$ (74)

Here *Z*_ref_ is a diagonal matrix whose elements are the $Z_{\mathrm{ref}}^{i}$ and U is another diagonal matrix defined by

U≡diag(|υ0i|υ0iRe(Zrefi)|Zrefi|). (75)

The factor U, which does not appear in other expressions relating S with *Z* [3,4], generalizes the earlier results to problems including complex fields and reference impedances.

Appendix D demonstrates that the off-diagonal elements of *Z* are related by

$$\frac{Z_{ji}}{Z_{ij}}=\frac{K_{i}}{K_{j}}\frac{\upsilon_{0_{i}}*}{\upsilon_{0_{i}}}\frac{\upsilon_{0j}}{\upsilon_{0j}*}.$$ (76)

Thus *Z*, like S, is generally asymmetric, even when the circuit is reciprocal and *v*_0_ is chosen real at each port. The asymmetry of *Z* is *not* a result of wave normalization, for *Z* is defined without reference to waves.

The admittance matrix Y is the inverse of *Z* and satisfies

$$i=Z^{-1}v=\mathrm{Yv}.$$ (77)

### 3.7 Change of Reference Impedance

As discussed earlier, the most convenient choice of reference impedance depends on the circumstances. In order to accommodate the various choices, we consider the relationship between the pseudo-wave amplitudes based on different reference impedances. By expressing $a\left(Z_{\mathrm{ref}}^{n}\right)$ and $b\left(Z_{\mathrm{ref}}^{n}\right)$ in terms of *v* and *i* using Eqs. (53) and (54) and *v* and *i* in terms of $a\left(Z_{\mathrm{ref}}^{m}\right)$ and $b\left(Z_{\mathrm{ref}}^{m}\right)$ using Eqs. (55) and (56), we arrive at the linear relationship

$$\left[\begin{array}{c} a\left(Z_{\mathrm{ref}}^{n}\right)\\ b\left(Z_{\mathrm{ref}}^{n}\right) \end{array}\right]=Q^{nm}\left[\begin{array}{c} a\left(Z_{\mathrm{ref}}^{m}\right)\\ b\left(Z_{\mathrm{ref}}^{m}\right) \end{array}\right],$$ (78)

where

Qnm≡12Zrefm|ZrefmZrefn|Re(Zrefn)Re(Zrefm).[Zrefm+ZrefnZrefm−ZrefnZrefm−ZrefnZrefm+Zrefn]. (79)

This can be put into more conventional form by defining a quantity *N_nm_*, analogous to the “turns ratio” of a conventional transformer, by

$$N_{nm}\equiv \sqrt{\frac{Z_{\mathrm{ref}}^{n}}{Z_{\mathrm{ref}}^{m}}},$$ (80)

so Eq. (78) becomes

Qnm≡12|Nnm|2Re(Zrefn)Re(Zrefm)[1+Nnm21−Nnm21−Nnm21+Nnm2]. (81)

Equation (81) is similar to the two-port cascade matrix of a classical impedance transformer [4], in which the square root in Eq. (81) is replaced by $N_{nm}^{*}$. When $Z_{\mathrm{ref}}^{m}$ and $Z_{\mathrm{ref}}^{n}$ are both real, the two matrices are identical. However, Eq. (81) can be determined neither from the classical result nor from any other lossless analysis. This explains why the result Eq. (79) does not, to our knowledge, appear in previous literature. Equations (78) and (79) are an exact expression of the *complex* impedance transform. We may accurately refer to the pseudo-waves as impedance-transformed traveling waves.

Two consecutive transforms can be represented as a single transform from the initial to the final reference impedance by

$$Q^{nm}Q^{mp}=Q^{np}.$$ (82)

Also,

$$Q^{nn}=I,$$ (83)

where I is the identity matrix. As a result,

$${\left[Q^{nm}\right]}^{-1}=Q^{mn},$$ (84)

which states that the transformation is inverted by a return to the original reference impedance.

The determinant of Q*^nm^* is

det[Qnm]=[1−jIm(Zrefm)Re(Zrefm)][1−jIm(Zrefn)Re(Zrefn)]−1. (85)

The scattering matrix associated with Q*^nm^* is symmetric if and only if det[Q*^nm^*] = 1, which is true if and only if the phases of $Z_{\mathrm{ref}}^{m}$ and $Z_{\mathrm{ref}}^{n}$ are identical. Equation (85) demonstrates that the scattering matrix representing the transform between a complex and a real impedance is in general asymmetric. In other words, a symmetric scattering matrix cannot remain symmetric when the reference impedance at a single port changes from a real to a nonreal value. This result is closely related to Eq. (64) since, from Eq. (69), the determinant of a cascade matrix is equal to *S*_12_/*S*_21_ of the associated scattering matrix S.

Q*^nm^* can be expressed in yet another form:

Qnm=1−jIm(Zrefm)/Re(Zrefm)1−jIm(Zrefn)/Re(Zrefn).11−Γnm2[1ΓnmΓnm1], (86)

where we use the definition

$$\Gamma_{nm}\equiv \frac{Z_{\mathrm{ref}}^{m}-Z_{\mathrm{ref}}^{n}}{Z_{\mathrm{ref}}^{m}+Z_{\mathrm{ref}}^{n}}.$$ (87)

This form is convenient in the computation of the effect of the complex impedance transform on the reflection coefficient. The reflection coefficient is transformed by

$$\Gamma \left(Z_{\mathrm{ref}}^{n}\right)=\frac{\Gamma_{nm}+\Gamma \left(Z_{\mathrm{ref}}^{m}\right)}{1+\Gamma_{nm}\Gamma \left(Z_{\mathrm{ref}}^{m}\right)}.$$ (88)

A short circuit, defined as a perfectly conducting electric wall spanning the entire cross section of the waveguide, forces the tangential electric field to vanish at the reference plane. A short therefore requires *v* = 0 and *b* = −*a.* As a result, the reflection coefficient is *Γ*_0_= −1. We can see from Eq. (88) that the transform of a perfect short remains $\Gamma \left(Z_{\mathrm{ref}}^{n}\right)=-1$, independent of the reference impedance. The only other reflection coefficient which is independent of the reference impedance is the perfect open circuit (magnetic wall), at which the transverse magnetic field vanishes so that *i* = 0, *b =a*, and *Γ*= +1. The unique status of the short and open is related to their unique physical manifestations.

If $\Gamma \left(Z_{\mathrm{ref}}^{m}\right)=0$ (perfect match) then $\Gamma \left(Z_{\mathrm{ref}}^{n}\right)=\Gamma_{nm}$. Conversely, if $\Gamma \left(Z_{\mathrm{ref}}^{m}\right)=-\Gamma_{nm}$ then $\Gamma \left(Z_{\mathrm{ref}}^{n}\right)=0$.

### 3.8 Multiport Reference Impedance Transformations

A direct, if somewhat complicated, means of computing the transformation of S due to a change of reference impedance begins by computing *Z* using Eq. (74). Subsequently, Eq. (73) is used with the *new* reference impedance to calculate the transformed S. This procedure works because *Z* is independent of reference impedance.

If the circuit under consideration is a two-port, the simplest way of computing the transform is to compute the associated cascade matrix R, perform the transform on R, and convert back to an S matrix. To determine the effect of the transform on R, we insert Eq. (78) into the right hand side of Eq. (68). In order to do the same with the left hand side, we need use the result that, due to symmetry of Q*^nm^* about both diagonals, Eq. (78) implies that

$$\left[\begin{array}{c} b\left(Z_{\mathrm{ref}}^{n}\right)\\ a\left(Z_{\mathrm{ref}}^{n}\right) \end{array}\right]=Q^{nq}\left[\begin{array}{c} b\left(Z_{\mathrm{ref}}^{q}\right)\\ a\left(Z_{\mathrm{ref}}^{q}\right) \end{array}\right].$$ (89)

Upon making these replacements and using Eq. (84), we can put Eq. (68) into a form relating $b_{1}\left(Z_{\mathrm{ref}}^{p}\right)$ and $a_{1}\left(Z_{\mathrm{ref}}^{p}\right)$ to $b_{2}\left(Z_{\mathrm{ref}}^{q}\right)$ and $a_{2}\left(Z_{\mathrm{ref}}^{q}\right)$. The result is that

$$R^{pq}=Q^{pm}R^{mn}Q^{nq}.$$ (90)

This equation displays the effect on the cascade matrix of altering the reference impedance of port 1 from $Z_{\mathrm{ref}}^{m}$ to $Z_{\mathrm{ref}}^{p}$ and that of port 2 from $Z_{\mathrm{ref}}^{n}$ to $Z_{\mathrm{ref}}^{q}$. This is a concise expression of the complex impedance transform.

In the special but common case in which the two ports use identical reference impedances, Eq. (90) simplifies. In transforming the reference impedance of both ports from $Z_{\mathrm{ref}}^{m}$ to $Z_{\mathrm{ref}}^{p}$, the cascade matrix is transformed by

$$\begin{array}{l} \;R^{pp}=Q^{pm}R^{mm}Q^{mp}=\\ \frac{1}{1-\Gamma_{pm}^{2}}\left[\begin{array}{cc} 1 & \Gamma_{pm}\\ \Gamma_{pm} & 1 \end{array}\right]R^{mm}\left[\begin{array}{cc} 1 & -\Gamma_{pm}\\ -\Gamma_{pm} & 1 \end{array}\right]. \end{array}$$ (91)

This transformation was used in Ref. [16].

### 3.9 Load Impedance

The load impedance is defined as the single element of the impedance matrix describing a linear one-port. At the reference plane, at which only a single mode exists, the load impedance is defined in terms of *v* and *i* as

$$Z_{\mathrm{load}}\equiv \frac{\upsilon }{i}.$$ (92)

From Eq. (19), the power absorbed by the load can be expressed as

$$P={\left|i\right|}^{2}R_{\mathrm{load}}={\left|\upsilon \right|}^{2}\frac{R_{\mathrm{load}}}{{\left|Z_{\mathrm{load}}\right|}^{2}},$$ (93)

where *R*_load_ ≡ Re(*Z*_load_). Power conservation ensures that, for a passive one-port, *R*_load_⩾0. For the remainder of this section, we assume that the load of interest is passive in order to avoid conflict with the requirement that Re(*Z*_ref_) ⩾0.

The load impedance, like *v* and *i*, is independent of the reference impedance. Unlike the result of low-frequency circuit theory, however, *Z*_load_ is *not* a unique property of the one-port itself but instead depends on the fields of the mode incident upon it. Illumination of the same device by a different waveguide, or even a different mode of the same waveguide, may result in a drastically different *Z*_load_. *Z*_load_ also depends on the normalization which determines *v*_0_ and *i*_0_, for this affects *v* and *i*.

Using Eq. (92) in Eq. (54), we see that, when the reference impedance is equal to the load impedance, we have *b* (*Z*_load_) = 0. From Eq. (58), this implies that

$$\Gamma \left(Z_{\mathrm{load}}\right)=0.$$ (94)

In other words, when *Z*_ref_ = *Z*_load_, the reflection coefficient vanishes. In this reference impedance, the load looks like a perfect match. Likewise, if we *insist* that the reflection coefficient vanishes when a certain load is connected to our line, we have effectively chosen the reference impedance to be equal to *Z*_load_ This is relevant to the calibration problem considered below. Keep in mind, however, that it may be difficult to establish a value for *Z*_load_ since that depends on the waveguide as well as the load.

Using Eq. (94) along with Eqs. (87) and (88), we find that

$$\Gamma \left(Z_{\mathrm{ref}}\right)=\frac{Z_{\mathrm{load}}-Z_{\mathrm{ref}}}{Z_{\mathrm{load}}+Z_{\mathrm{ref}}}.$$ (95)

We can also solve for *Z*_load_:

$$Z_{\mathrm{load}}=Z_{\mathrm{ref}}\frac{1+\Gamma \left(Z_{\mathrm{ref}}\right)}{1-\Gamma \left(Z_{\mathrm{ref}}\right)}.$$ (96)

This produces the same result regardless of the reference impedance with respect to which *Γ* is defined. If we choose *Z*_ref_ equal to the characteristic impedance *Z*_0_, these two equations are identical to those of ordinary waveguide circuit theory and to the theory of Ref. [6].

We see from Eq. (96) that the load impedance of a short is 0 and that of an open is ∞.

As an example of a load, consider the use of a semi-infinite transmission line with characteristic impedance *Z*_1_ to terminate a transmission line with characteristic impedance *Z*_0_. In general, the reflection coefficient and the load impedance are impossible to compute. One common approximation, based on the notions of low-frequency circuit theory, is that both *v* and *i* are continuous at the interface. This assumption leads to the result that the load impedance of the line is simply its characteristic impedance. This allows the reflection coefficient to be determined by Eq. (95).

Unfortunately, the assumption leading to this result is not generally valid, since *v* and *i* are not generally continuous at an interface. Recall that *v* and *i* are not strictly related to true voltage or current. The actual boundary conditions at the interface require continuity of tangential fields, and these cannot in general be satisfied without the presence of an infinity of higher order modes at the discontinuity. By contrast, the waveguide voltage and current are indicative of the intensities of only a *single* mode. The reflection coefficient cannot therefore be determined from waveguide circuit parameters. For an explicit example, consider the case in which *Z*_0_= *Z*_1_ while the two transmission lines are physically dissimilar. In this case, the assumption that the load impedance equals *Z*_1_ leads to the result that there is no reflection of traveling waves. In fact, reflection *must* take place due to the discontinuity at the interface. Exceptions occur only when no higher-order modes are generated. An example is coaxial lines of lossless conductors which differ only in the dielectric material. In this peculiar example, the reflection coefficient can be computed exactly from *Z*_0_ and *Z*_1_. In other examples, the result is at best approximate.

## 4. Waveguide Metrology

In this section, we apply the theoretical results of the previous sections to the elucidation of the basic problems of waveguide metrology, which aims to characterize waveguide circuits in terms of appropriate matrix descriptions.

### 4.1 Measurability and the Choice of Reference Impedance

In addition to the slotted line, which measures VSWR directly, the primary instrument used to characterize waveguide circuits is the vector network analyzer (VNA). Here we restrict ourselves to a two-port VNA, which provides a measurement M*_i_* of the product

$$M_{i}={\mathrm{XT}}_{i}\bar{Y}.$$ (97)

Here T*_i_* is the cascade matrix of the device *i* under test, X and Y are constant, non-singular matrices which describe the instrument, and

$$\bar{Y}\equiv \left[\begin{array}{cc} 0 & 1\\ 1 & 0 \end{array}\right]Y^{-1}\left[\begin{array}{cc} 0 & 1\\ 1 & 0 \end{array}\right]$$ (98)

is the reverse cascade matrix corresponding to Y. The problem of network analyzer calibration is to determine X and Y by the insertion and measurement of known devices *i.* With X and Y known, Eq. (97) determines T*_i_* from the measured M*_i_*.

X, Y, and T*_i_* are commonly considered unique, and a calibration process which determines them uniquely is applied. However, as we have seen in this paper, the cascade matrix T*_i_* depends on the reference impedances with which it is defined. Thus, any number of calibrations lead to legitimate measurements of a cascade matrix and therefore legitimate measurements of pseudo-scattering parameters, although with varying port reference impedances. We refer to these calibrations, each of which is related to any other by an impedance transform, as *consistent.* Any calibration which is not related to a consistent calibration by an impedance transform will not yield measurements of pseudo-scattering parameters. Such a calibration is *inconsistent.* For example, X and Y may be determined in such a way that the resulting measurement of an open circuit is not equal to 1. Such a result is prohibited for pseudo-scattering parameters, so the calibration is inconsistent. It is meaningless to speak of the reference impedance of such a calibration.

The reference impedances of a consistently calibrated VNA are uniquely determined by the calibration. Only when the reference impedance is equal to the characteristic impedance of the line are the resulting pseudo-scattering parameters equal to the actual scattering parameters. Of course, transformation to an alternative reference impedance is possible, but only if the initial reference impedance is *known.* This section analyzes some common calibration methods to determine their reference impedance.

We assume that the waveguides at the two reference planes and the two corresponding basis functions *e_t_* are identical. When *Z*_ref_ at both ports is equal to the characteristic impedance *Z*_0_, we can express Eq. (97) as

$$M_{i}=X^{0}T_{i}^{0}{\bar{Y}}^{0}.$$ (99)

The single superscript on the network analyzer matrices refers to the reference impedance at the test ports. We do not need to define or discuss a reference impedance at the “measurement ports.”

From Eq. (84), the identity matrix can be expressed as I=Q^0^*^m^*Q*^m^*^0^ Inserting this into Eq. (99) yields

$$M_{i}=\left(X^{0}Q^{0m}\right)\left(Q^{m0}T_{i}^{0}Q^{0n}\right)\left(Q^{n0}{\bar{Y}}^{0}\right)=X^{m}T_{i}^{mn}{\bar{Y}}^{n},$$ (100)

where

$$X^{m}\equiv X^{0}Q^{0m},$$ (101)

$${\bar{Y}}^{n}\equiv Q^{n0}{\bar{Y}}^{0},$$ (102)

and

$$T_{i}^{mn}\equiv Q^{m0}T_{i}^{0}Q^{0n}$$ (103)

are the impedance-transformed cascade matrices. If the calibration procedure determines that X=X*^m^* and Y=Y*^n^*, then subsequent calibrated measurements will determine the matrix $T_{i}^{mn}$. If X*^m^ and* Y*^m^* have the form of Eqs. (101) and (102), the VNA will be consistently calibrated to reference impedances $Z_{\mathrm{ref}}^{m}$ on port 1 and $Z_{\mathrm{ref}}^{n}$ on port 2.

The most accurate method of VNA calibration is TRL [17, 18], a moniker which refers to the use of a “thru,” and “reflect,” and a “line.” The “thru” is a length of transmission line which connects at either end to a test port. The line standard is a longer section of transmission line. The “reflect” is a symmetric and transmissionless but otherwise arbitrary two-port embedded in a section of transmission line. The method assumes that each measured device has an identical transition from the test port to the calibration reference plane. The reference planes are set to the center of the thru.

The TRL method, like other calibration methods, determines the matrices X*^m^* and Y*^n^*. However, as we have seen, these two matrices are nonunique since they depend on the reference impedances. Thus, we need to analyze the algorithm to determine which reference impedances are imposed by the calibration.

Our first standard (*i* = 1), an ideal thru, is a continuous connection between two identical lines. Since the traveling waves are not disturbed, the cascade matrix using a reference impedance of *Z*_0_ must be the identity matrix I:

$$T_{1}^{0}=I.$$ (104)

If the calibration is consistent but, instead of *Z*_0_, reference impedances $Z_{\mathrm{ref}}^{m}$ and $Z_{\mathrm{ref}}^{n}$ are used, then the thru has the cascade matrix

$$T_{1}^{mn}=Q^{m0}|Q^{0n}=Q^{mn}.$$ (105)

However, the TRL algorithm is constructed so as to force the calibrated measurement of the thru to equal the identity matrix. That is, it imposes the condition that

$$T_{1}^{mn}=Q^{mn}=I,$$ (106)

which, from (86) and (87), is true if and only if

$$Z_{\mathrm{ref}}^{m}=Z_{\mathrm{ref}}^{n}.$$ (107)

In other words, the algorithm imposes the condition that the reference impedances on both ports be identical. The thru alone cannot provide any information as the value of that reference impedance.

Another result of the TRL algorithm is that the calibrated measurement of the reflect standard is identical on both ports. This again reveals nothing about the port reference impedances except that they are identical.

The ideal line standard (*i* = 2) is a length of transmission line identical to that of the two test ports and connected to them without discontinuity. As a result, there is no reflection of the traveling waves. This requires the cascade matrix of the line, with a reference impedance of *Z*_0_, to be

$$T_{2}^{0}=\left[\begin{array}{cc} e^{-\gamma l} & 0\\ 0 & e^{+\gamma l} \end{array}\right],$$ (108)

where *γ* is the propagation constant and *l* is the line length. Since we require identical reference impedances on both ports, the transformed cascade matrix is

$$\begin{array}{c} T_{2}^{mm}=Q^{m0}T_{2}^{0}Q^{0m}=\\ \frac{e^{+\gamma l}}{1-\Gamma_{0m}^{2}}\left[\begin{array}{cc} e^{-2\gamma l}-\Gamma_{0m}^{2} & \left(1-e^{-2\gamma l}\right)\Gamma_{0m}\\ -\left(1-e^{-2\gamma l}\right)\Gamma_{0m} & 1-e^{-2\gamma l}\Gamma_{0m}^{2} \end{array}\right], \end{array}$$ (109)

where *Γ*_0m_ is defined as in Eq. (87).

The TRL algorithm ensures that the cascade matrix in Eq. (109) is diagonal and therefore that the calibrated measurement of the line will be such that *S*_11_=*S*_22_ = 0. The off-diagonal elements of (109) are equal and opposite. Assuming that $e^{-2\gamma l}\neq 1,\;T_{2}^{mm}$ is diagonal if and only if *Γ*_0m_ =0, which implies that Q^0^*^m^* = I and

$$Z_{\mathrm{ref}}^{m}=Z_{0}.$$ (110)

That is, the TRL method using a perfect line and thru results in a consistent calibration with identical reference impedances on each port equal to the characteristic impedance of the line. Recall that the condition *Z*_ref_*=Z*_0_ was the condition under which the pseudo-waves are equal to the actual traveling waves. Thus *the TRL method calibrates the VNA so as to measure the unique scattering matrix* S^0^ *which relates the actual traveling waves*, not some arbitrary pseudo-scattering matrix S.

In the special case *e^−^*^2^*^γl^*= 1, as occurs in a lossless line whose phase delay is an integral multiple of 180°, T*^mm^* is diagonal for *any Γ*_0_*_m_*. Therefore, the reference impedance need not be equal to *Z*_0_ and is in fact indeterminate. This results in the well-known problem of ill-conditioning in such a case.

We have seen that the TRL method calibrates to a reference impedance of *Z*_0_. What happens if we use the TRL *algorithm* but not the TRL *standards*? We consider methods which use the thru and reflect but replace the ideal line by some other passive artifact, which we call the surrogate line. The matrix $T_{2}^{0}$ takes the arbitrary form

$$T_{2}^{0}\equiv \left[\begin{array}{cc} A & B\\ C & D \end{array}\right].$$ (111)

Since the use of the thru forces any consistent calibration to have identical reference impedances on each port, the transformation of $T_{2}^{0}$ is

$$T_{2}^{mm}=\frac{1}{\sqrt{1-\Gamma_{0m}^{2}}}\;\left[\begin{array}{c} A+B\Gamma_{0m}-C\Gamma_{0m}-D\Gamma_{0m}^{2}+A\Gamma_{0m}+B-C\Gamma_{0m}^{2}-D\Gamma_{0m}\\ -A\Gamma_{0m}-B\Gamma_{0m}^{2}+C+D\Gamma_{0m}-A\Gamma_{0m}^{2}-B\Gamma_{0m}+C\Gamma_{0m}+D \end{array}\right].$$ (112)

The algorithm attempts to force $T_{2}^{mm}$ to be diagonal. With a surrogate in place of the line, this may be impossible if $T_{2}^{mm}$ has the form of Eq. (112), for we have two equations to be satisfied but only the single variable *Γ*_0_*_m_*. The sum of those two equations produces the requirement

C=−B, (113)

which is identical to the condition

$$S_{11}^{0}=S_{22}^{0}$$ (114)

on the scattering parameters of the standard.

Unless Eq. (114) is satisfied, the analysis reveals a contradiction. The resolution of this problem lies with the realization that Eq. (112) results from the assumption that the calibration is *consistent.* However, unless Eq. (114) is satisfied, the calibration is *inconsistent* and Eq. (112) does not apply. This conclusion is almost obvious, given the fact that both the thru and the surrogate line must appear perfectly matched at each port. In order to meet this condition with a consistent calibration, the thru requires identical reference impedances on each port while the surrogate line demands different reference impedances. Consequently, the calibration is inconsistent and no reference impedance exists.

Clearly, the perfect line meets the symmetry criterion (114). However, so do many other artifacts. Given standards that satisfy (114), a consistent calibration is obtained and the condition of diagonality determines *Γ*_0_*_m_*. When *B* = *C* = 0, as was the case with the TRL method, then *Γ*_0_*_m_* = 0 and the reference impedance is *Z*_0_. In any other case, *Γ*_0_*_m_* is determined by a quadratic equation whose solution is

$$\Gamma_{0m}=\frac{D-A}{2B}\pm \sqrt{{\left[\frac{D-A}{2B}\right]}^{2}-1.}$$ (115)

The cascade parameters *A, B, C*, and *D* can be replaced by the scattering parameters of the standard:

$$\frac{D-A}{B}=S_{11}^{0}+\frac{1}{S_{11}^{0}}-\frac{S_{12}^{0}S_{21}^{0}}{S_{11}^{0}}.$$ (116)

This formally determines the reference impedance, albeit in a somewhat complicated fashion. In the special case $S_{12}^{0}S_{21}^{0}=0$, the insertion of Eq. (116) into (115) leads to the two solutions $\Gamma_{0m}=S_{11}^{0}$ and $\Gamma_{0m}=1/S_{11}^{0}$. An analysis lets us reject the second of these. It is then simple to show that

$$Z_{\mathrm{ref}}^{m}=Z_{\mathrm{load}}.$$ (117)

That is, the reference impedance for the calibration is the load impedance of the device used as a standard. As indicated by Eq. (94), this is the appropriate reference impedance so that the calibrated reflection coefficient vanishes.

Since the standard is assumed passive, then, from Eq. (93), Re(*Z*_load_)⩾0. Therefore, Eq. (117) presents no conflicts with the requirement that Re(*Z*_ref_)⩾=0.

This sort of calibration is known as TRM or LRM [19], where the “M” stands for “match.” Clearly, the match need not be perfect. If the match *is* perfect $\left(S_{11}^{0}=S_{22}^{0}=0\right)$, then the calibration is identical to that using TRL and will allow the measurement of relations between traveling waves. If the match is symmetric but imperfect and $S_{12}^{0}=S_{21}^{0}=0$, *the LRM calibration is related to the TRL calibration by an impedance transform of both ports to a reference impedance equal to the load impedance of the match.* In this case, the VNA calibrated with LRM measures relations not among the traveling waves but among a particular set of pseudo-waves.

Frequently, the match standard is chosen to be a pair of small resistors in the hope that their load impedance is approximately real and constant. This would lead to a useful calibration in which the pseudo-scattering parameters would be measured with respect to a real, constant reference impedance. Unfortunately, it is difficult in practice to design a real and constant load impedance. Furthermore, that impedance is known only after it has been measured with respect to some other calibration. In addition, the load impedance generally depends on the line with respect to which it is measured.

If $S_{11}^{0}=S_{22}^{0}\neq 0$ and $S_{12}^{0}=S_{21}^{0}\neq 0$, as would be the case using a symmetric attenuator, the calibration reference impedance depends on $S_{12}^{0}=S_{21}^{0}$ as well as $S_{11}^{0}$ of the standard. This is an important point to consider in designing the match standard, for any coupling between the two resistors will induce a shift in the reference impedance compared to the load impedance of either resistor alone.

Another useful example is the mismatched line standard. The TRL method using an ideal, matched line led to a reference impedance equal to the characteristic impedance of the line. Since this perfect line is identical to the line at the test port, the traveling waves are not reflected. What happens if the line standard, while uniform, is *not* identical to the test port? The problem is similar to one described in the previous section. In general, the question is impossible to answer. However, for illustration, we consider the approximation that *v* and *i* are continuous at the interface. In this case, we can compute the cascade matrix of the line of characteristic impedance *Z_i_* as

$$T_{2}^{0}=\;\frac{e^{+\gamma l}}{1-\Gamma_{0l}^{2}}\left[\begin{array}{cc} e^{-2\gamma l}-\Gamma_{0l}^{2} & \left(1-e^{-2\gamma l}\right)\Gamma_{0l}\\ -\left(1-e^{-2\gamma l}\right)\Gamma_{0l} & 1-e^{-2\gamma l}\Gamma_{0l}^{2} \end{array}\right],$$ (118)

which can be transformed to

$$T_{2}^{mm}=\;\frac{e^{+\gamma l}}{1-\Gamma_{ml}^{2}}\left[\begin{array}{cc} e^{-2\gamma l}-\Gamma_{ml}^{2} & \left(1-e^{-2\gamma l}\right)\Gamma_{ml}\\ -\left(1-e^{-2\gamma l}\right)\Gamma_{ml} & 1-e^{-2\gamma l}\Gamma_{ml}^{2} \end{array}\right].$$ (119)

This is identical in form to the previous result for a perfect line standard. It leads to the result

$$Z_{\mathrm{ref}}^{m}=Z_{l}.$$ (120)

In this approximation, the reference impedance is the characteristic impedance of the line. This potentially useful result suggests that a particular line may be used as a calibration standard for any network analyzer with identical results. However, the assumption that *v* and *i* are continuous, which led to the result, is not generally valid. The example of a 50 Ω, 2.4 mm coaxial standard used on 50 Ω, 3.5 mm coaxial test ports makes this clear, for the standard must reflect the traveling waves even though its characteristic impedance is appropriate for a reflectionless standard. In general, the quality of the approximation depends in detail on the nature of the waveguide interface.

Calibration using any of these devices, as long as $S_{11}^{0}=S_{22}^{0}$, leads to solutions differing only by a change of reference impedance. Of course, we can easily transform between any two reference impedances if given the values. A procedure to transform between LRL and LRM calibrations [16] is based on measuring the load reflection coefficient with respect to an LRL calibration. However, this is only a *relative* transformation; the initial and final reference impedances remain unknown. The most comprehensive procedure is to determine the absolute *Z*_ref_. A method to accomplish this combines the TRL calibration using a nominally perfect line with a measurement of *Z*_0_, which in this case is identical to *Z*_ref_ [12]. It is difficult to imagine determining the reference impedance of any of the other calibration methods, even in principle, without comparison to a TRL calibration.

Many calibration methods other than those based on the TRL algorithm are in use. These typically require the measurement of artifacts, such as open and short circuits, whose scattering parameters are presumed known. Although electromagnetic simulations may provide good estimates, the actual scattering parameters can be known accurately only by measurement. Thus the calibration artifacts must be viewed as transfer standards. If the scattering parameters are given incorrectly, the calibration may be inconsistent. However, if perfect short and open circuits are used along with a termination defined as a perfect match, it is possible to obtain a consistent calibration with the reference impedance equal to the load impedance of the termination.

### 4.2 Measurement of Pseudo-Waves and Waveguide Voltage and Current

The methods of the previous section provide for the measurement of ratios of pseudo-waves. In order to measure the wave amplitudes, an additional magnitude measurement is necessary. The most convenient parameter to measure is the power *P.* From measurements of *P* and *Γ* and a known *Z*ref, Eq. (59) allows the determination of |*a*|. This applies to |*a*_0_| as well if we replace *Z*_ref_ by *Z*_0_. The absolute phases of the pseudo-waves and traveling waves cannot be measured without specifying the arbitrary phase of the modal fields. However, the relative phase of *a* and *b* is given by Eq. (58).

Once *a* and *b* have been determined, |*v*| and |*i*| are given by Eqs. (55) and (56). The ratio of these two equations determines the relative phase of *v* and *i.*

## 5. Alternative Circuit Theory Using Power Waves

In addition to the pseudo-waves *a* and *b* defined by Eqs. (53) and (54), other quantities may be defined using different linear combinations of *v* and *i.* Popular alternatives are the “incident and reflected wave amplitudes” normalized to “complex port numbers” [7]. For a complex port number $\hat{Z}$, these quantities are defined by

a^(Z^)≡ν+iZ^2Re(Z^) (121)

and

b^(Z^)≡ν−iZ^*2Re(Z^). (122)

In Ref. [7], $\hat{Z}$ is arbitrary except that Re(Z^)>0; this restriction is lifted in subsequent publications. When $\hat{Z}$ is the load impedance of the device connected to the port, *a* and *b* are known as power waves [8]. For simplicity, we shall use the term “power waves” for all quantities of the form (121) and (122).

We take *v* and *i* to be the waveguide voltage and current defined in Sec. 2. Like Ref. [7], we limit our discussion to the case Re(Z^)>0.

**Fig. 6:**
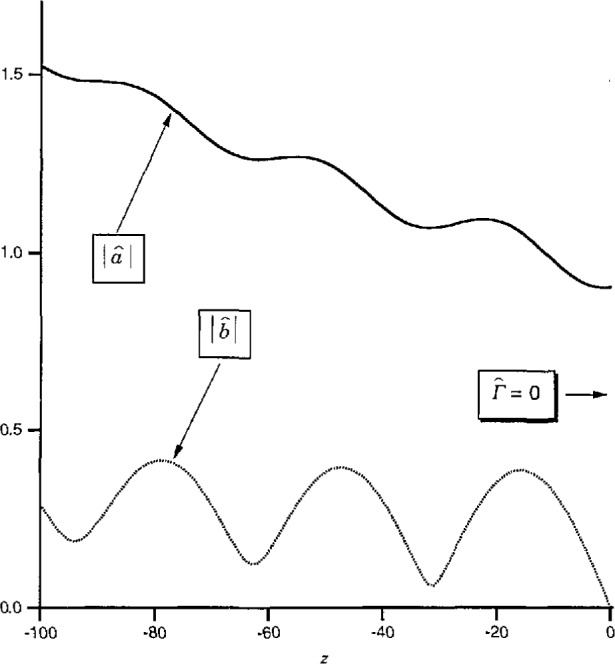
The magnitudes of the power waves *â* and $\hat{b}$ for the example of Fig. 4. The characteristic impedance is taken to be 1–0.2*j*. $\hat{Z}$ is chosen so that $\hat{\Gamma }(\hat{Z})$vanishes at the termination reference plane._ Since the waves depend in a complicated fashion on z, $\hat{\Gamma }(\hat{Z})$ vanishes *only* at *z* = 0.

When $\hat{Z}$ is real, the power waves reduce to pseudo-waves (except for a phase factor) with reference impedance $Z_{\mathrm{ref}}=\hat{Z}$. Otherwise they do not correspond. The power waves are *not* equal to the traveling waves for *any* choice of $\hat{Z}$ unless the characteristic impedance is real. For example, Fig. 6 plots the power wave magnitudes corresponding to the example of Fig. 4; $\hat{Z}$ is chosen so that $\hat{b}$ vanishes at *z* = 0. This figure illustrates that the power waves are complicated functions of *z;* it is clearly unrealistic to interpret them as “incident and reflected waves.”

The power waves are devised to satisfy the simple power relation

$$p=|\hat{a}{|}^{2}-|\hat{b}{|}^{2}$$ (123)

for any $\hat{Z}$. The pseudo-waves satisfy a relationship of this form only when *Z*_ref_ is real.

Power wave scattering parameters can be defined analogously to the pseudo-scattering parameters. For example, the power wave reflection coefficient is

$$\hat{\Gamma }(\hat{Z})\equiv \frac{\hat{b}(\hat{Z})}{\hat{a}(\hat{Z})}=\frac{\nu -i\hat{Z}*}{\nu +i\hat{Z}}=\frac{Z_{\mathrm{load}}-\hat{Z}*}{Z_{\mathrm{load}}+\hat{Z}},$$ (124)

which should be contrasted to Eq. (95). The power wave reflection coefficient of an open circuit (*i* = 0) is equal to 1, the same as the pseudo-wave reflection coefficient defined earlier. However, the result for a short circuit (*v* = 0) is

$$\nu =0\Rightarrow \hat{\Gamma }(\hat{Z})=-\frac{\hat{Z}*}{\hat{Z}},$$ (125)

which is equal to the pseudo-wave reflection coefficient −1 only in the special case Im(Z^)=0. This indicates clearly that the power waves are not generally related to the traveling waves by an impedance transform.

The implications of this are significant. For instance, the relationship between the load impedance and the pseudo-reflection coefficient is given by Eq. (95), which is the classical result. It is the basis of the Smith chart as well as most circuit design software. On the other hand, the equivalent relationship in terms of power wave quantities is Eq. (124), to which the Smith chart does not apply since it does not represent a linear fractional transformation. To sharpen this distinction, recall that the Smith chart is based on a *normalized* impedance; that is, the load impedance displayed on the chart is relative to *Z*_ref_ (*Z*_0_ in the case of traveling waves). The chart is able to accommodate the data in this form because the pseudo-reflection coefficient, as illustrated by Eq. (95), depends only on the *ratio Z*_load_*/Z*_ref_. The power wave reflection coefficient, however, depends not only on the ratio $Z_{\mathrm{load}}/\hat{Z}$ but also on the phase of $\hat{Z}$. Therefore, an attempt to generalize the Smith chart to display power wave reflection coefficients must lead to a *separate* chart for each phase of $\hat{Z}$.

Recall that the pseudo-wave scattering matrix of a reciprocal circuit is not generally symmetric in lossy waveguides. In contrast, advocates of power waves argue that the power wave scattering matrix of a lossy, reciprocal circuit is symmetric. For waveguide circuits, this is false. The usual derivation of symmetry makes use of the symmetry of the impedance matrix, which, as we have seen, does not hold for waveguides. Thus, one ubiquitous justification of a power wave description of waveguide circuits is invalid. The correct reciprocity relationship is given in Appendix D.

Although a complete circuit theory based on power waves is possible, we have chosen not to develop one, for several reasons. Unlike the power waves, the pseudo-waves are related to the traveling waves by an impedance transform and therefore avoid the problems discussed above. Furthermore, unlike the power waves, the pseudo-waves can generally be set equal to the traveling waves by an appropriate choice of the reference impedance. Although the pseudo-waves do not generally satisfy a simple power expression of the form Eq. (123), they can always be made to do so by an appropriate choice of the reference impedance. Typically this involves choosing *Z*_ref_ to be real, but the choice of *Z*_ref_=*Z*_load_, analogous to the choice $\hat{Z}=Z_{\mathrm{load}}$ made by Ref. [8], will also suffice.

Although a network analyzer may be used to measure power waves, such a use is rare for, as illustrated in the previous section, it is the pseudo-waves that are measured using conventional calibration techniques. None of these methods may be easily modified to directly measure power waves. Methods which apply shorts and opens as calibration standards are inapplicable since only the open, not the short, is a useful power wave standard. Furthermore, the TRL method cannot be applied to power wave measurement since it is closely tied to the measurement of traveling waves.

One method of measuring a power wave reflection coefficient begins with first measuring the pseudo-wave reflection coefficient. If the reference impedance of that calibration can be determined, then the load impedance may be calculated from Eq. (96); the power wave reflection coefficient can then be determined from Eq. (124). Methods which do not require the determination of the pseudo-wave parameters as a prerequisite appear to be unknown at this time. In any case, such methods do *not* exist in the firmware which controls conventional network analyzers, so that these machines are incapable of determining power wave scattering parameters without external software.

## Footnotes

1 The magnitude of the complex fields was defined to be the root-mean-square, rather than the peak, of the time-dependent fields. This accounts for the absence of the factor 1/2 in the expression for the power.

## 6. Appendix A. Reduction of Maxwell’s Equations

The electric and magnetic fields of a mode have been designated *ee^−γz^* and *he^−γz^.* For the moment, we will allow anisotropy and therefore introduce the tensor permittivity ***ϵ*** and tensor permeability ***μ***. Maxwell’s equations take the form

$$\nabla \times (ee^{-\gamma z})=-j\omega \mu \cdot (he^{-yz}),$$ (A1)

$$\nabla \times (he^{-\gamma z})=+j\;\omega \;\epsilon \cdot (ee^{-yz}),$$ (A2)

$$\nabla \cdot (\epsilon \cdot ee^{-\gamma z})=0,$$ (A3)

and

$$\nabla \cdot \left(\mu \cdot he^{-\gamma z}\right)=0,$$ (A4)

which readily reduce to

∇×e−γz×e=−jωμ⋅h, (A5)

∇×h−γz×h=+jωϵ⋅e, (A6)

∇⋅(ϵ⋅e)=γ(ϵ⋅e)⋅z, (A7)

and

∇⋅(μ⋅h)=γ(μ⋅h)⋅z. (A8)

If we now divide *e* and *h* into their transverse and axial components, Eqs. (A5) and (A6) become

$$\nabla \times e_{t}=-j\omega \left(\mu \cdot h\right)\cdot z,$$ (A9)

$$\nabla \times h_{t}=+j\omega \left(\epsilon \cdot e\right)\cdot z$$ (A10)

$$z\times \nabla e_{z}+\gamma z\times e_{t}=+j\omega {\left(\mu \cdot h\right)}_{t},$$ (A11)

and

$$z\times \nabla h_{z}+gz\times h_{t}=-j\omega {\left(\epsilon \cdot e\right)}_{t}.$$ (A12)

For the isotropic materials discussed in the text, Eqs. (A7)–(A12) reduce to Eqs. (2)–(7). In general, it appears difficult to generalize the text to include materials of arbitrary anisotropy. However, generalization is fairly simple in the absence of terms in *ϵ* and *μ* coupling between transverse and axial field components. In that case, we can write

$$\epsilon =\epsilon_{t}+\epsilon_{z}zz;\;z\cdot \epsilon_{t}=\epsilon_{t}\cdot z=0$$ (A13)

and

$$\mu =\mu_{t}+\mu_{z}zz;\;z\cdot \mu_{t}=\mu_{t}\cdot z=0.$$ (A14)

All of the results in the text follow with slight modification. For example, equations Eqs. (B5) and (B6), from which the circuit parameter expressions arise, must be modified by the following replacements:

$$\epsilon {\left|e_{t}\right|}^{2}\to {e_{t}}^{*}\cdot \epsilon_{t}\cdot e_{t},$$ (A15)

$$\mu {\left|h_{t}\right|}^{2}\to {h_{t}}^{*}\cdot \mu_{t}\cdot h_{t},$$ (A16)

$$\epsilon {\left|e_{z}\right|}^{2}\to \epsilon_{z}{\left|e_{z}\right|}^{2},$$ (A17)

and

$$\mu {\left|h_{z}\right|}^{2}\to \mu_{z}{\left|h_{z}\right|}^{2}.$$ (A18)

## 7. Appendix B. Circuit Parameter Integral Expressions

Taking the scalar product of both sides of Eq. (5) with z*×e_t_^*^* results in

$$\begin{array}{r} \gamma z\cdot {e_{t}}^{*}\times h_{t}+z\cdot {e_{t}}^{*}\times \nabla h_{z}=\\ +j\omega \epsilon \left(z\times {e_{t}}^{*}\right)\cdot \left(z\times e_{t}\right)=+j\omega \epsilon {\left|e_{t}\right|}^{2}. \end{array}$$ (B1)

Integrating over the cross section of the waveguide and recognizing the first integral as *p*_0_^*^ = |υ_0_|^2^/*Z*_0_, we have

$$\frac{\gamma }{Z_{0}}=\frac{1}{{\left|\upsilon_{0}\right|}^{2}}\left[j\omega \int_{S}\epsilon {\left|e_{t}\right|}^{2}dS-z\cdot \int_{S}{e_{t}}^{*}\times \nabla h_{2}dS\right].$$ (B2)

The second integral can be manipulated into a simpler form. First apply Stokes’s Law to the vector *h_z_e_t_^*^* to yield

$$\begin{array}{l} \int_{S}\nabla \times \left(h_{z}{e_{t}}^{*}\right)\cdot zdS=\int_{S}h_{z}\nabla \times {e_{t}}^{*}\cdot zdS-\\ \int_{S}{e_{t}}^{*}\times \nabla h_{z}\cdot zdS=\int_{\partial S}h_{z}{e_{t}}^{*}\cdot dl, \end{array}$$ (B3)

where ∂*S* is the boundary of *S* and d*l* is a line element along that boundary. If the waveguide is transversely closed by a perfectly conducting boundary, then *S* coincides with that boundary and the line integral vanishes. If the waveguide is open, then a portion of *S* may lie at infinity, but the integral also vanishes as long as *e_t_* vanishes fast enough to ensure that the modal power is finite. Finally, although Stokes’ Law cannot formally be applied across material discontinuities, it can readily be shown that the line integrals on both sides of the boundary are equal and opposite. As a result, the line integral in Eq. (B3) vanishes. The insertion of Eq. (2) simplifies Eq. (B3) to

$$\begin{array}{c} z\cdot \int_{S}{e_{t}}^{*}\times \nabla h_{z}dS=\int_{S}h_{z}\nabla \times {e_{t}}^{*}\cdot zdS=\\ j\omega \int_{S}\mu^{*}{\left|h_{z}\right|}^{2}dS, \end{array}$$ (B4)

so Eq. (B2) becomes

$$\frac{\gamma }{Z_{0}}=\frac{j\omega }{{\left|\upsilon_{0}\right|}^{2}}\left[\int_{S}\epsilon {\left|e_{t}\right|}^{2}dS-\int_{S}\mu^{*}{\left|h_{z}\right|}^{2}dS\right].$$ (B5)

By an analogous procedure using Eqs. (3) and (4), we may demonstrate that

$$\gamma Z_{0}=\frac{j\omega }{{\left|i_{0}\right|}^{2}}\left[\int_{S}\mu {\left|h_{t}\right|}^{2}dS-\int_{S}\epsilon^{*}{\left|e_{z}\right|}^{2}dS\right].$$ (B6)

The use of Eqs. (B5) and (B6) along with definitions (29) and (30) results in Eqs. (33)–(36) for the circuit parameters *C*, *L*, *G*, and *R.*

## 8. Appendix C. Relations Between *p*_0_ and *γ*

From Eqs. (20), (29), and (30),

$$\gamma {p_{0}}^{*}={\left|\upsilon_{0}\right|}^{2}\left[j\omega C+G\right]$$ (C1)

and

$$\gamma p_{0}={\left|i_{0}\right|}^{2}\left[j\omega L+R\right],$$ (C2)

from which it can readily be shown that

2Re(γ)Re(p0)=+|υ0|2G+|i0|2R, (C3)

2Re(γ)Im(p0)=−|υ0|2ωC+|i0|2ωL, (C4)

2Im(γ)Re(p0)=+|υ0|2ωC+|i0|2ωL, (C5)

and

2Im(γ)Im(p0)=+|υ0|2G−|i0|2R. (C6)

An interesting alternative form of Eq. (C5) is

Re(p0)=ωβ[12|υ0|2C+12|i0|2L]. (C7)

This is the real average power carried by the forward mode at *z* = 0. For TEM modes, it is the product of the group velocity *ω*/*β* and the energy density (per unit length), represented by the term in brackets.

If the materials are lossless, then certain useful results apply. In that case, |*v*_0_|^2^*G* = |*i*_0_|^2^*R* = 0. Aside from the degenerate case in which *γp*_0_ = 0, only two sorts of modes may exist. The first, which we denote propagating modes, satisfy

Re(γ)=Im(p0)=Im(Z0)=0;Im(γ)≠0;Re(p0)>0, (C8)

which implies that they propagate without decay with a real characteristic impedance. Equation (C4) becomes

(Re(γ)=0)⇒|υ0|2C=|i0|2L, (C9)

leaving free only one of the four parameters *R*, *C*, *G*, and *L*. Equation (C9) can be expanded as

(Re(γ)=0)⇒∫Sμ|h|2dS=∫Sϵ|e|2dS. (C10)

This states the well-known result [3] that the energy in a lossless propagating wave is divided equally between the electric and magnetic fields.

Modes in lossless media with *γp*_0_≠0 that are *not* propagating satisfy

Im(γ)=Re(p0)=Re(Z0)=0;Re(γ)>0;Im(p0)≠0, (C11)

and therefore

$${\left|\upsilon_{0}\right|}^{2}C=-{\left|i_{0}\right|}^{2}L.$$ (C12)

These modes are purely evanescent, decaying exponentially and, in isolation, carrying no real power. The inductance and capacitance are of opposite sign.

If we restrict ourselves to passive but not necessarily lossless media, certain converse results apply. Passivity ensures that *G* and *R* are nonnegative. Thus, if either Re(*γ*) = 0 or *Re*(*p*_0_) = 0, then Eq. (C3) requires |*v*_0_|^2^*G* = |*i*_0_|^2^*R* = 0. Since *ϵ″* and *μ″* are nonnegative in passive media, Eqs. (35) and (36) require that *ϵ″e = μ″h* = 0 everywhere. Now, if *e* = 0, then Maxwell’s equations imply that *h* = 0 (and *vice versa*), except in the case *ω* = 0, which we have explicitly excluded. Therefore, in passive media, the possibilities Re(*γ*) = 0 (unattenuated mode) or Re(*p*_0_) = 0 (mode carrying no real power) occur only if *ϵ″* = *μ″* = 0; that is, only when the media are lossless. In contrast, there is no apparent prohibition against Im(*γ*) or Im(*p*_0_) vanishing in lossy media.

Finally, we treat the degenerate modes in which either *γ* or *p*_0_ vanishes. From Eqs. (C1) and (C2), these modes satisfy

$$\left\{\gamma p_{0}=0\right\}\Rightarrow {\left|\upsilon_{0}\right|}^{2}C={\left|\upsilon_{0}\right|}^{2}G={\left|i_{0}\right|}^{2}L={\left|i_{0}\right|}^{2}R=0.$$ (C13)

The second and fourth conditions ensure that such degeneracy occurs only in lossless waveguides.

If *γ* = 0, then Maxwell’s equations decouple into one set [Eqs. (2), (5), and (6)] involving only ***e****_t_*, and *h_z_* and another set [Eqs. (3), (4), and (7)] involving only ***h****_t_*, and *e_z_.* Therefore, we can decompose the fields into modes with either ***e****_t_, =h_z_ = 0* or ***h****_t_=e_z_ = 0.* In the former case, |*v*_0_|^2^ *C* automatically vanishes, due to Eq. (33), and the condition |*i*_0_|^2^ *L* = 0 constrains the remaining fields; the opposite holds true in the latter case. In either situation, *p*_0_ = 0 since the Poynting vector ***e****_i_* × ***h****_i_* vanishes. In this case *γ =p*_0_ = 0, exemplified by a lossless waveguide mode operating exactly at the cutoff frequency, the forward and backward modes are indistinguishable.

On the other hand, *p*_0_ = 0 does *not* imply that *γ* = 0. Furthermore, in contrast to the lossless case with *p*_0_ ≠ 0 discussed above, *γ* is *not* restricted to be real or imaginary. “Complex waves,” in which *γ* is neither real nor imaginary even though the materials are lossless, have been discovered in inhomogeneous as well as in anisotropic media. They are discussed in Ref. [20] and references included therein.

## 9. Appendix D. Reciprocity Relations

Consider two sets of electromagnetic fields (***E***′, ***H***′) and (***E***″, ***H***″), which are produced by two different sets of boundary conditions. Applying the divergence theorem to *E*′ *× H*″ and using the homogeneous Maxwell’s equations produces the well-known result that

$$\int \left(E^{\prime }\times H^{\prime \prime }-E^{\prime \prime }\times H^{\prime }\right)\cdot n\;dS=0,$$ (Dl)

whenever the permittivity and permeability tensors are symmetric. In Eq. (D1), the surface encloses a closed region and the unit vector ***n*** is the outward normal to the surface. We let the surface enclose an entire waveguide junction and become infinitely large in such a way that the contributions to the integral can be entirely accounted for by the single mode of interest propagating in each waveguide leaving the junction. Expressing the fields in each port ***n*** in terms of Eqs. (12) and (13), Eq. (D1) becomes

$$\sum_{n}\frac{{\upsilon_{n}}^{\prime }{i_{n}}^{\prime \prime }}{\upsilon_{0n}i_{0n}}{\tilde{p}}_{0n}=\sum_{n}\frac{{\upsilon_{n}}^{\prime \prime }{i_{n}}^{\prime }}{\upsilon_{0n}i_{0n}}{\tilde{p}}_{0n},$$ (D2)

having defined

$${\tilde{p}}_{0n}\equiv \int_{S_{n}}e_{in}\times h_{in}\cdot z\;dS,$$ (D3)

where *S_n_* is the cross section of the *n*th waveguide. Equation (D2) can be written as the matrix equation

$${i^{\prime \prime }}^{t}{Wv^{\prime }=v^{\prime \prime }}^{t}Wi^{\prime }.$$ (D4)

As before, i and v are column vectors of *i_n_* and *v_n_*, and “t” stands for “transpose.” W is the diagonal matrix

$$W\equiv \mathrm{diag}\left(W_{n}\right);W_{n}\equiv \frac{{\tilde{p}}_{0n}}{\upsilon_{0n}i_{0n}}\equiv \frac{\upsilon_{0n}^{*}}{\upsilon_{0n}}\frac{{\tilde{p}}_{0n}}{{\tilde{p}}_{0n}^{*}},$$ (D5)

where Eq. (20) has been used. Inserting V = Zi into Eq. (D4) and requiring that the result holds for all values of i′ and i″, we determine that

$$Z^{t}={\mathrm{WZW}}^{-1},$$ (D6)

which is the reciprocity requirement on the impedance matrix. It requires that the elements of *Z* satisfy

$$Z_{nm}=\frac{W_{m}}{W_{n}}Z_{mn}.$$ (D7)

To determine the analogous condition on S, take the transpose of Eq. (E5):

$$S^{t}=U^{-1}{\left(Z^{t}+Z_{\mathrm{ref}}\right)}^{-1}\left(Z^{t}-Z_{\mathrm{ref}}\right)U.$$ (D8)

Insert Eq. (D6) and factor out W and W^−1^, noting that W *Z*_ref_ W^−1^= *Z*_ref_ since W and *Z*_ref_ are diagonal. The result is

$$S^{t}=U^{-1}W{\left(Z+Z_{\mathrm{ref}}\right)}^{-1}\left(Z-Z_{\mathrm{ref}}\right)W^{-1}U.$$ (D9)

The two central terms can be commuted using the fact that any matrices A and B satisfy

$${\left(A+B\right)}^{-1}\left(A-B\right)=B^{-1}\left(A-B\right){\left(A+B\right)}^{-1}B$$ (D10)

as long as the inverses exist. Using Eq. (D10) in Eq. (D9) and using Eq. (E5) to express the result in terms of S, we have

$$S^{t}=P^{-1}\mathrm{SP},$$ (D11)

using the definition

$$P\equiv Z_{\mathrm{ref}}U^{2}W^{-1}.$$ (D12)

Since P is diagonal, Eq. (D11) requires that the elements of S satisfy

$$S_{nm}=\frac{P_{nn}}{P_{mm}}S_{mn},$$ (D13)

which is expressed more explicitly as Eq. (64) of the text.

We can also develop a reciprocity relation for the power wave scattering matrix, defined by

$$\hat{b}=\hat{S}\hat{a},$$ (D14)

where

$$\hat{a}=F\left(v+\hat{Z}i\right)$$ (D15)

and

$$\hat{b}=F\left(v-\hat{Z}*i\right)$$ (D16)

are the vector forms of Eqs. (121) and (122). We have defined

$$\hat{Z}\equiv \mathrm{diag}\left(\hat{Z}\right)$$ (D17)

and

$$F\equiv \mathrm{diag}\left(\frac{1}{2\sqrt{\hat{Z}}}\right).$$ (D18)

Inserting Eqs. (D15) and (D16), as well as v = Zi, into Eq. (D14) and insisting that the result hold for all i yields

$$\hat{S}=F\left(Z-\hat{Z}*\right){\left(Z+\hat{Z}\right)}^{-1}F^{-1},$$ (D19)

the transpose of which is

$${\hat{S}}^{t}=F^{-1}{\left(Z^{t}+\hat{Z}\right)}^{-1}\left(Z^{t}-\hat{Z}*\right)F.$$ (D20)

Using Eq. (D6) and some simple manipulation leads to

$${\hat{S}}^{t}=F^{-1}W{\left(Z+\hat{Z}\right)}^{-1}\left(Z-\hat{Z}*\right)W^{-1}F.$$ (D21)

Reference [8] shows that

$$\begin{array}{c} {\left(Z+\hat{Z}\right)}^{-1}\left(Z-\hat{Z}*\right)=\\ F^{2}\left(Z-\hat{Z}*\right){\left(Z+\hat{Z}\right)}^{-1}F^{-2}=F\hat{S}F^{-1}, \end{array}$$ (D22)

so that Eq. (D21) reduces to the simple result

$${\hat{S}}^{t}={W\hat{S}W}^{-1}$$ (D23)

The power wave scattering matrix therefore obeys a reciprocity relation identical to the one (D6) satisfied by the impedance matrix. In lossy waveguides, neither is generally symmetric.

## 10. Appendix E. Relations Between Z and S

Recall that a, b, v, and i are defined as column vectors whose elements are *a_m_*, *b_m_*, *v_m_*, and *i_m_* at the various waveguide ports *m*. The vector representation of Eqs. (53) and (54) are

$$a=\frac{1}{2}U\left(v+Z_{\mathrm{ref}}i\right)$$ (E1)

and

$$b=\frac{1}{2}U\left(v-Z_{\mathrm{ref}}i\right),$$ (E2)

where U is a diagonal matrix defined by

U≡diag(|υ0m|υ0mRe(Zrefm)Zrefm). (E3)

Inserting v = Zi into Eqs. (E1) and (E2) eliminates v. The condition b = Sa then implies

$$b=\frac{1}{2}U\left(Z-Z_{\mathrm{ref}}\right)i=\mathrm{Sa}=\frac{1}{2}\mathrm{SU}\left(Z+Z_{\mathrm{ref}}\right)i.$$ (E4)

Since this must hold for all i, we can solve for S, yielding

$$\begin{array}{l} S=U\left(Z-Z_{\mathrm{ref}}\right){\left(Z+Z_{\mathrm{ref}}\right)}^{-1}U^{-1}=\\ U\left({\mathrm{ZZ}}_{\mathrm{ref}}^{-1}-1\right){\left({\mathrm{ZZ}}_{\mathrm{ref}}^{-1}+1\right)}^{-1}U^{-1}. \end{array}$$ (E5)

This can be easily inverted to produce

$$Z={\left(I-U^{-1}\mathrm{SU}\right)}^{-1}\left(1+U^{-1}\mathrm{SU}\right)Z_{\mathrm{ref}}.$$ (E6)

## 11. Appendix F. Renormalization Table

The text allows for the arbitrarily normalization of the parameters ***e****_t_* and ***v***_0_. This table details the effects of renormalizing these two parameters on the remaining variables. The second column shows the effect on the element in the first column of multiplying ***e****_t_* by the factor *α.* The third column shows the results of a change in the voltage integration path which multiplies *v*_0_ by the factor *β*. No result is shown if the variable is independent of the normalization.

**Table:**

Renormalization table		

*γ*		
***E**_t_,**H**_t_*		
***e**_t_, **h**_t_*	***αe**_t_*, ***αh**_t_*	
***C***_+_,***C***_−_	c_+_/*α*, c_−_/*α*	
*v*_0_	*αv*_0_	*βv*_0_
*i*_0_	*αi*_0_	*i*_0_*/β**
*v*		*βv*
*i*		*i/β**
*p*_0_	|*α*|^2^*p*_0_	
*p*		
*p*		
*Z*_0_		|*β*|^2^*Z*_0_
*C, G*		*C/|β*|^2^, *G/|β*|^2^
*L, R*		|*β*|^2^*L*, |*β*|^2^*R*
*α*_0_, *b*_0_	$\frac{\left|\alpha \right|}{\alpha }a_{0},\frac{\left|\alpha \right|}{\alpha }b_{0}$	
*a*(*Z*_ref_), *b*(*Z*_ref_)	$\frac{\left|\alpha \right|}{\alpha }a_{0}\left(Z_{\mathrm{ref}}\right),\frac{\left|\alpha \right|}{\alpha }b\left(Z_{\mathrm{ref}}\right)$	$a\left(Z_{\mathrm{ref}/{\left|\beta \right|}^{2}}\right),b\left(Z_{\mathrm{ref}/{\left|\beta \right|}^{2}}\right)$
${\tilde{p}}_{0}$	$\alpha^{2}{\tilde{p}}_{0}$	
*K_n_*	$\left(\frac{\alpha }{\left|\alpha \right|}\right)2\;K_{n}$	
***W****_n_*		$\frac{\beta *}{\beta }W_{n}$
$S_{ij}^{0}$	$\frac{\left|\alpha_{i}\right|}{\alpha_{i}}\frac{\alpha_{j}}{\left|\alpha_{j}\right|}S_{ij}^{0}$	
$S_{ij}\left(Z_{\mathrm{ref}}^{i},Z_{\mathrm{ref}}^{j}\right)$	$\frac{\left|\alpha_{i}\right|}{\alpha_{i}}\frac{\alpha_{j}}{\left|\alpha_{j}\right|}S_{ij}\left(Z_{\mathrm{ref}}^{i},Z_{\mathrm{ref}}^{j}\right)$	$S_{ij}(Z_{\mathrm{ref}}^{i}{/}_{{\left|\beta_{i}\right|}^{2}},Z_{\mathrm{ref}}^{j}{/}_{{\left|\beta_{j}\right|}^{2}})$
*Γ*_0_		
*Γ*(*Z*_ref_)		$\Gamma (Z_{\mathrm{ref}}{/}_{{\left|\beta \right|}^{2}})$
*Z_ij_*		*β_i_ β_j_* *Z*_ij_*
*Z*_load_		|*β*|^2^ *Z*_load_
*Y_ij_*		*Y_ij_*/(*β*_i_* *β_j_*)
**R**^0^	$\frac{\left|\alpha_{1}\right|}{\alpha_{1}}\frac{\alpha_{2}}{\left|\alpha_{2}\right|}R^{0}$	
$R\left(Z_{\mathrm{ref}}^{1},Z_{\mathrm{ref}}^{2}\right)$	$\frac{\left|\alpha_{1}\right|}{\alpha_{1}}\frac{\alpha_{2}}{\left|\alpha_{2}\right|}R\left(Z_{\mathrm{ref}}^{1},Z_{\mathrm{ref}}^{2}\right)$	$R(Z_{\mathrm{ref}}^{1}{/}_{{\left|\beta_{1}\right|}^{2}},Z_{\mathrm{ref}}^{2}{/}_{{\left|\beta_{2}\right|}^{2}})$
